# Structural analysis of influenza vaccine virus-like particles reveals a multicomponent organization

**DOI:** 10.1038/s41598-018-28700-7

**Published:** 2018-07-09

**Authors:** Dustin M. McCraw, John R. Gallagher, Udana Torian, Mallory L. Myers, Michael T. Conlon, Neetu M. Gulati, Audray K. Harris

**Affiliations:** Laboratory of Infectious Diseases, National Institute of Allergy and Infectious Diseases, National Institutes of Health, 50 South Drive, Room 6351, Bethesda, MD 20892 USA

## Abstract

Influenza virus continues to be a major health problem due to the continually changing immunodominant head regions of the major surface glycoprotein, hemagglutinin (HA). However, some emerging vaccine platforms designed by biotechnology efforts, such as recombinant influenza virus-like particles (VLPs) have been shown to elicit protective antibodies to antigenically different influenza viruses. Here, using biochemical analyses and cryo-electron microscopy methods coupled to image analysis, we report the composition and 3D structural organization of influenza VLPs of the 1918 pandemic influenza virus. HA molecules were uniformly distributed on the VLP surfaces and the conformation of HA was in a prefusion state. Moreover, HA could be bound by antibody targeting conserved epitopes in the stem region of HA. Taken together, our analysis suggests structural parameters that may be important for VLP biotechnology such as a multi-component organization with (i) an outer component consisting of prefusion HA spikes on the surfaces, (ii) a VLP membrane with HA distribution permitting stem epitope display, and (iii) internal structural components.

## Introduction

Enveloped viruses, such as influenza, infect millions of people world-wide on an annual basis. During the 1918 pandemic the influenza virus killed 50 to 100 million people^1–4^. The major influenza surface glycoprotein, hemagglutinin (HA), mediates viral entry by undergoing a prefusion to postfusion conformational change and some broadly neutralizing antibodies target conserved epitopes on stem regions displayed only on the prefusion state of HA^5,6^. Although the use of structure-guided design approaches is gaining more attention in efforts to evaluate epitope conformation and display with the goal to improve the immunogenicity and efficacy of nanoparticles for a number of viral systems^7–9^, efforts to invoke structure-guided design of membrane containing vaccine VLPs that are pleiomorphic in structure has not been studied to a great extent. This may be because many nanoparticle platforms are refractory to structural techniques, such as X-ray crystallography, due to possible sample flexibility and pleiomorphy that impede crystallization. Here, we refer to nanoparticles as particles without a membrane and virus-like particles (VLPs) as nanoparticles with a membrane. Cryo-electron microscopy (Cryo-EM) is a structural biology technique in which structures with membranes, flexibility and pleiomorphy can be studied^10^. Due to the pleiomorphy of influenza virus, it has been studied by cryo-EM methods^11–13^.

However, the 3D structural organization of influenza vaccine VLPs has not been addressed to a large extent nor has comparison between vaccine VLPs and virus particles. Thus, the structure guided-design and characterization of influenza VLPs has not been explored in great detail. Lack of such information on the 3D molecular structure of influenza VLPs hinders studies on the role of particle organization on facilitating and stabilizing HA epitope display for optimizing membrane-containing immunogens for influenza vaccines. Here, we focused on biochemical and cryo-electron microscopy analysis of recombinant H1 HA VLPs because this HA subtype represents viruses that have caused 1918 and 2009 influenza pandemics. Also, in previous studies, these VLPs produced very broadly protective responses including to challenge with lethal doses of influenza viruses expressing different HA subtypes^14^.

We report that VLPs were dominated by spherical morphologies that were similar in sizes to influenza virions. The VLPs could be generalized as being constructed of multiple structural components. HA spike components were projecting from the VLP surfaces and were embedded in the membrane component. HA molecules appeared to be in a prefusion conformation and immunoassays with antibodies indicated epitope display and integrity of conserved stem epitopes. Also, VLPs contained internal component material. In terms of overall future prospects, our findings suggest that the structure-guided evaluation and design of influenza HA VLPs could be used to optimize the design of VLPs to carry immune factors or other influenza antigens. Such designed VLPs could promote broadly protective immune responses to different strains and subtypes of seasonal and pandemic influenza viruses. In addition, influenza VLPs could be systems used to elucidate general design principles of VLP platforms for biotechnology vaccine efforts focused on viral glycoproteins.

## Results

### Analysis of VLP proteins

**Figure 1:**
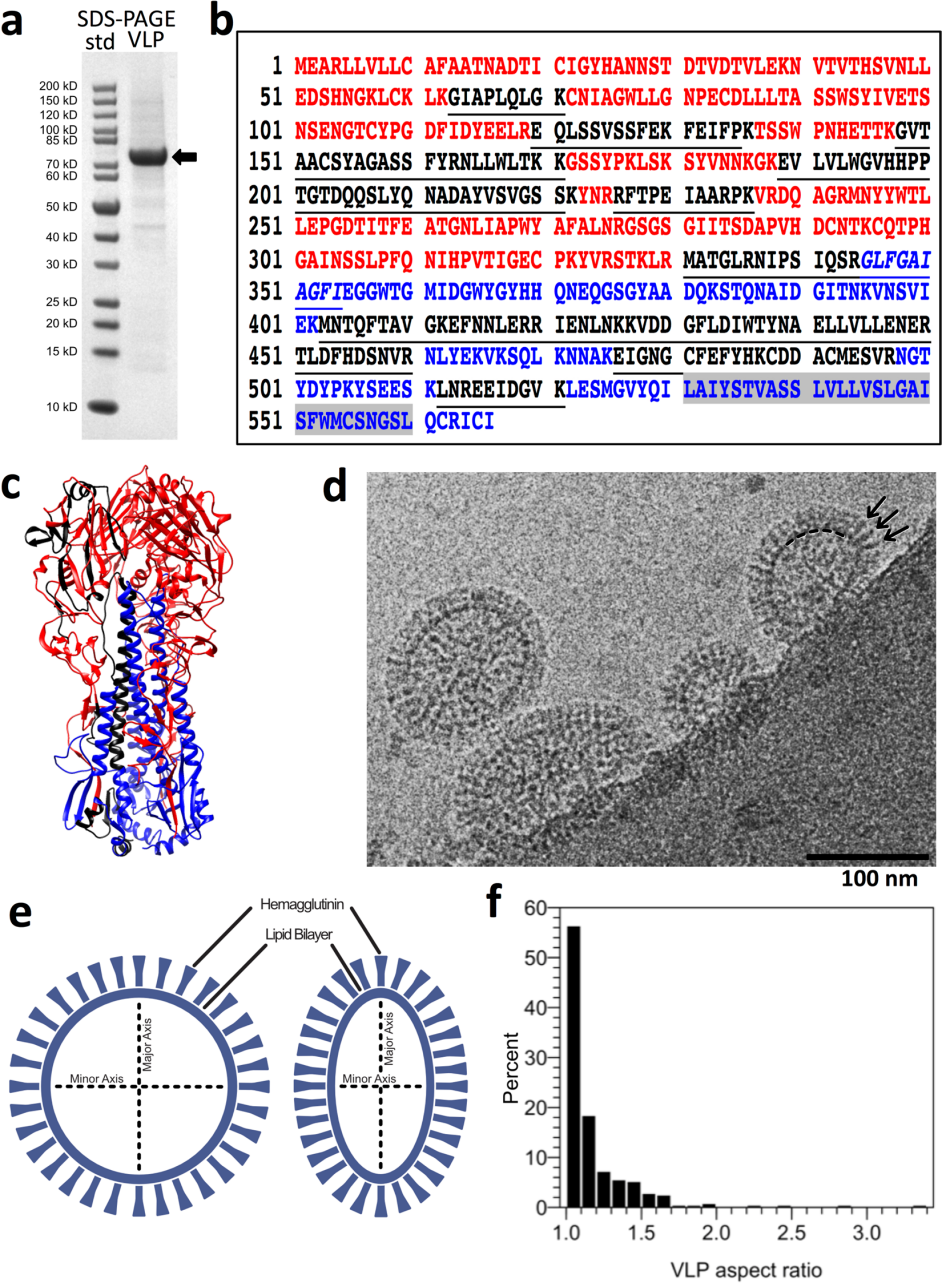
Analysis of composition and organization of 1918 hemagglutinin (HA) H1 virus-like particles (VLPs) by peptide-finger printing and cryo-electron coupled with axial measurements. (**a**) SDS-PAGE analysis of VLPs under reducing conditions. Molecular weight standards (std) are in lane 1 and the VLP sample is in lane 2. An arrow denotes a band at an apparent molecular weight of about 75 kilodaltons. (**b**) Peptide finger printing of the major 75 kDa protein band of VLPs by mass spectrometry of tryptic peptides with database query of peptide profile matched to hemagglutinin sequence (A/Brevig Mission/1/1918 (H1N1)) by mass spectrometry with 35% coverage. The HA1 region sequence (M1-R344) is colored red with the HA2 region (G345-I566) in blue. The transmembrane region of HA2 is highlighted in grey. However, matched HA peptide regions are colored black within the sequence. (**c**) Peptides (black) mapped on the crystal structure of H1 HA from 1918 (PDB ID 1RD8) with HA1 and HA2 regions in red and blue, respectively. For clarity, the peptides are only mapped onto one HA1-HA2 protomer on the left-side. (**d**) Image by cryo-electron microscopy of a field of VLPs. Arrows denote some protruding spikes on the surface on a particle. The particles have oval-shaped outlined perimeters at the base of the protruding spikes. A portion of this outline in one particle is denoted by a hashed arc near the base of the indicated spikes (right particle). Scale bar, 100 nm. (**e**) Schematics of spherical and elongated VLP morphologies defined with a system of two axes as minor (shorter) and major (longer) that are used to determine aspect ratios and lengths. The dark peripheral outline and the spikes observed in cryo images are schematically assigned to the lipid membrane and hemagglutinin, respectively. (**f**) Distribution of VLP aspect ratio for a population of VLPs (N = 295). The aspect ratio is the ratio between major and minor axes as measured in x and y directions from cryo-EM images. Based on aspect ratios VLPs were assigned as spherical (1.0–1.1), near spherical (>1.1 and <1.5) and elongated (>1.5).

1918 VLPs were produced by co-expression of influenza structural proteins, hemagglutinin (HA) H1 and matrix (M1) as previously reported^14^. In order to determine the relative purity and protein composition of VLPs, SDS-PAGE analysis and mass spectrometry of protein bands were performed. Also, to study the organization of VLPs, they were imaged by cryo-electron microscopy. Proteomic analysis revealed that hemagglutinin (HA) was the most abundant protein in the sample (Fig. 1a**–**c) and HA was on the surface of the VLPs and appeared as spikes (Fig. 1d). A major protein band with an apparent molecular weight of about 75 kDa was present (Fig. 1a). Based on the size, it was provisionally assigned to HA. Densitometry and subsequent one-dimensional profile analysis of the banding pattern indicated that the major band accounted for about 90% of the protein in the VLP sample (Fig. S1). Of note was the lack of detection of a band for the influenza matrix protein with an apparent molecular weight of about 28 KDa for analyzed influenza virus samples used for comparison to VLPs (Fig. S2b). Peptide fingerprinting of the major band of the VLPs by mass spectrometry identified the band to be HA of a H1 subtype. The mass spectrometry signature matched hemagglutinin from A/Brevig Mission/1/1918 with 35% coverage throughout both the HA1 and HA2 domains, confirming the identity as 1918 hemagglutinin (Fig. 1b,c). The major peptide matches by mass spectrometry were to hemagglutinin (Table S1). HA appeared to be the uncleaved HA0 form (~75 kDa) and not cleaved into HA1 and HA2.

Because hemagglutinin, matrix (M1), and nucleoprotein (NP) proteins comprise the majority of the influenza structural proteins (Fig. S2), we analyzed the HA-dominant VLPs by electron microscopy to determine if HA was on the surface and to what extent. Initial negative-stained electron microscopy indicated particles with spikes on the surfaces (Fig. S3). By negative-staining, VLPs did not display long filamentous morphologies like those observed for an H3 virus (A/Victoria/3/75) isolated from MDCK cells (Fig. S4a,b). While negative staining provides high-contrast, concern for staining artifacts persist because samples are chemically fixed and dehydrated. Thus, we used cryo-electron microscopy (cryo-EM) to further study the sample in which samples are in a frozen-hydrated state. In cryo-EM VLPs appeared as isolated particles with protruding spikes on the surfaces (Fig. 1d, arrows) emanating from a membrane density perimeter (Fig. 1d, dotted line). The surface appeared to have a dense covering of spikes (i.e. HA) on the surfaces and in some cases individual spikes could be resolved (Fig. 1d, arrows). The 1918 H1 HA VLPs, derived from a baculovirus expression system, exhibited a corona of surface spikes (Figs 1d, S5) similar to that observed for cryo-EM images of influenza viruses isolated from chicken eggs (Fig. S2a) and MDCK culture systems (Fig. S4c–f).

### Morphology and size of VLPs

**Figure 2:**
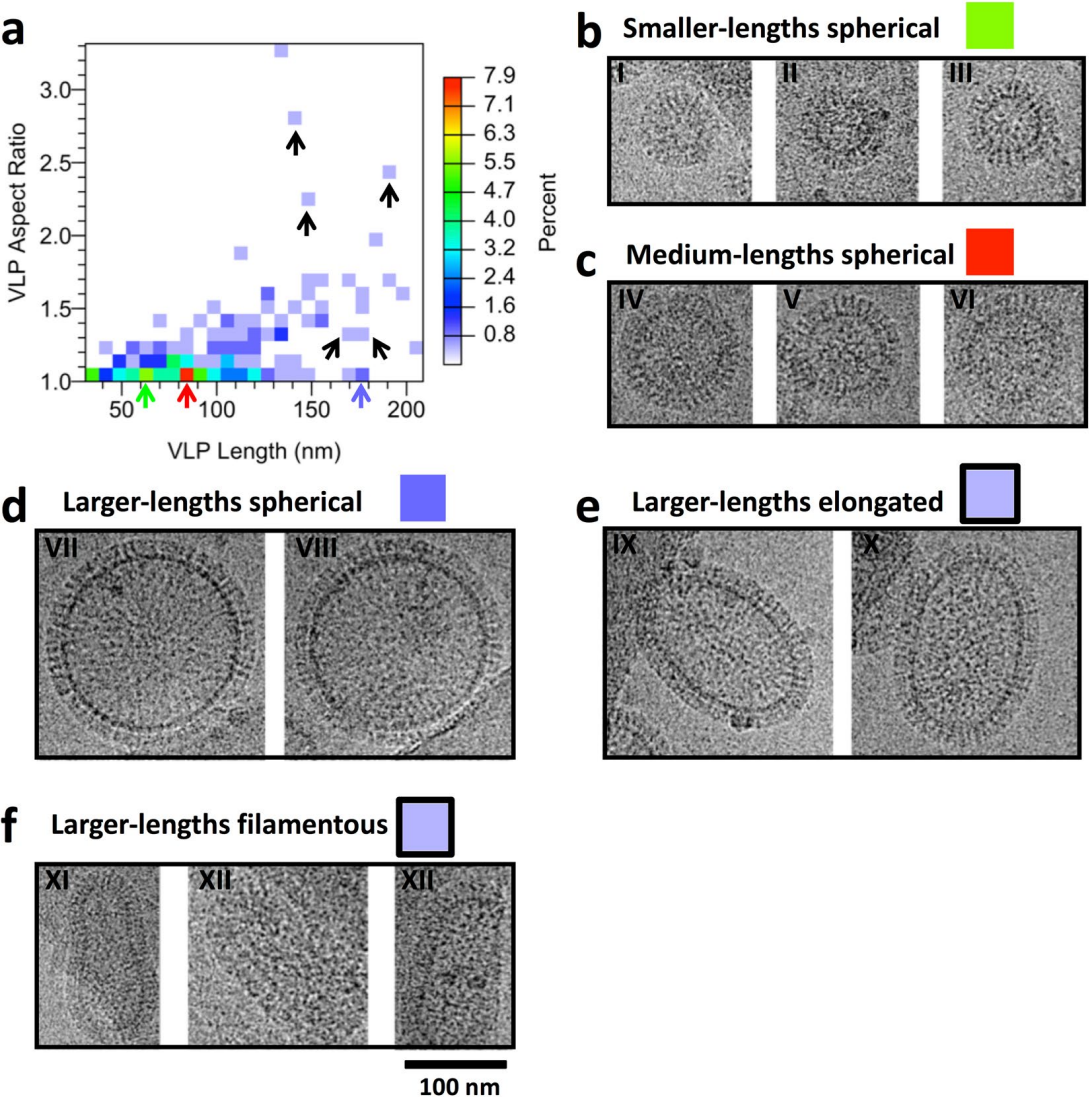
Analysis of VLP size and morphology by heat-map analysis with examples from groups with various lengths and aspect ratios as measured by cryo-electron microscopy. (**a**) A heat map of VLP aspect ratios plotted against VLP lengths, which are the major axes. By convention for an ellipse the major axis is the longest axis. The color scale is on the side. Areas of higher percentages are seen at about 60 nm length (green arrow) and 85 nm length (red arrow). The apparent majority of the VLPs approach spherical morphologies with aspect ratios less than 1.2. Some spherical VLPs can be larger in size (blue arrow). A smaller fraction of VLPs are larger in lengths and with larger aspect ratios that are in the heat-map as scattered dots (black arrows). (**b**) Examples of individual spherical VLPs with a length of about 60 nm (panels I-III). (**c**) Examples of individual spherical VLPs with a length of about 85 nm (panels IV–VI). (**d**) Examples of individual spherical VLPs with a length of about 170 nm (panels VII, VIII). VLPs in panel b, c, and d have aspect ratios less than 1.1. Lager spherical VLPs (Panel d) display VLPs with a length of about 170 nm. (**e**) Larger VLPs but with aspect ratio around 1.3 display and oval elongated morphology. (**f**) VLPs with aspect ratios greater than 2.2 appear filamentous in morphology. Green, red, and bluish squares denote the color regions from the heat map (panel a) where the VLPs were chosen with respect to length for spherical particles with aspect ratios less than 1.2. Elongated (panel e) and filamentous particles (panel f) are denoted by black arrows in the heat map (panel a). Panels b, c, d, e, and f are shown on the same scale. Scale bar, 100 nm.

Because influenza virus particles can have both spherical and filamentous morphologies (Figs S2, S4), the morphologies and sizes of VLPs were analyzed by cryo-electron microscopy to assess structural variation of the VLPs. We collected 2D cryo-EM images of VLPs (Figs 1d, S5) and characterized individual VLPs (N = 295) by both size and morphology. 2D cryo-EM allowed hundreds of VLP images to be collected for analysis. Analyses indicated that the majority of VLPs were spherical in shape (Figs 1e,f, 2). Although there was variation among the sizes of the particles, glycoprotein spikes (HA) were observed on the surfaces of all sizes and morphologies. When subsets of VLPs were arranged in order of largest to smallest as panel montages all size variations had HA spikes on the surfaces (Figs 2, S5). On visual inspection of 2D cryo-EM images, the VLP morphologies appeared to be dominated by spherically shaped particles. In order to quantitate the spherical nature of the VLPs, they were measured by the major and minor axis of an ellipse fit to the VLP membrane (Fig. 1e).

The ellipticity of VLPs was assessed by the ratio of the major axis to the minor axis (i.e. aspect ratio), where a circle is an ellipse with an aspect of ratio of 1. By convention for an ellipse the major axis is the longest axis. The average aspect ratio ranged from 1.0 to 3.3 across all VLPs. However, the VLP population had a distribution that was highly skewed towards 1.0 (Fig. 1f). The largest bin of VLPs (~56%) had aspect ratios from 1.0 to 1.1 and was categorized as spherical (Fig. 1f). The second largest bin had aspect ratios of 1.1 to 1.2 and was categorized as near spherical (~18%). Based on this distribution, about 75% of the particles were approaching spherical morphology (aspect ratio < 1.2: major axis = 82 ± 28 nm, minor axis = 78 ± 27 nm). The remaining 25% being judged as elongated based on histogram frequency (aspect ratio > = 1.2: major axis = 128 ± 41 nm, minor axis = 87 ± 26 nm). Across the entire population of VLPs, the mean value for the major axis was 94 ± 37 nm, and the minor axis was 80 ± 27 nm, and the mean aspect ratio was 1.17 ± 0.26.

### Heat-map analysis of particle size

The distribution of particle sizes and aspect ratios was visualized using heat-map analysis of the VLP lengths of the major axes versus the VLP aspect ratios (Fig. 2a). This was done to determine if VLP size correlated with ellipticity. We were then able to pick voxels from the heat-map analysis to inspect cryo images that contained representative VLPs of a given morphology (Fig. 2). The heat-map indicated a higher frequency of VLPs of spherical morphologies with a denser distribution clustering towards the bottom left side of the heat-map (Fig. 2a). VLPs from regions with aspect ratios of 1.0 to 1.1 (i.e. spherical) but with variable lengths along the major axis (VLP length) were compared by observing their organization with cryo-EM images. Particle populations at the bottom of the heat map (green, red and blue frequency regions) (Fig. 2a, green, red, and light-blue arrows, respectively) displayed rather uniform spherical shapes when their images were visually examined (Fig. 2b–d). Comparisons indicated spherically shaped groups having apparent smaller (Fig. 2b, I–III), medium (Fig. 2c, IV–VI), and larger lengths (Fig. 2d, VII–VIII). Particles in the green group had a size of about 60 nm (Fig. 2b), the red group was about 85 nm in size (Fig. 2c) and the light-blue group was larger at about 170 nm is size (Fig. 2d). In contrast particles that were found at both larger VLP lengths and aspect ratios were a lower percentage of the population (Fig. 2a, black arrows). Examination of VLP images from these regions indicated elongated (Fig. 2e) and filamentous morphologies (Fig. 2f). Thus, the VLP length range of 150 to 200 nm of the heat-map (Fig. 2a) coupled to VLP cryo images from this region indicated that increasing VLP aspect ratios matched with particle morphologies being elongated (oval-shaped) (Fig. 2e), and filamentous (Fig. 2f). It appeared that an aspect ratio greater than 2.2 gave a filamentous morphology. Nevertheless, despite differences in sizes and shapes, the surfaces of the VLPs from the different heat-map groups (Fig. 2b–f) appeared to be covered with HA glycoprotein spikes is a similar fashion. The glycoprotein HA spikes were further analyzed by image analysis.

### Image analysis of VLP surface spikes

**Figure 3:**
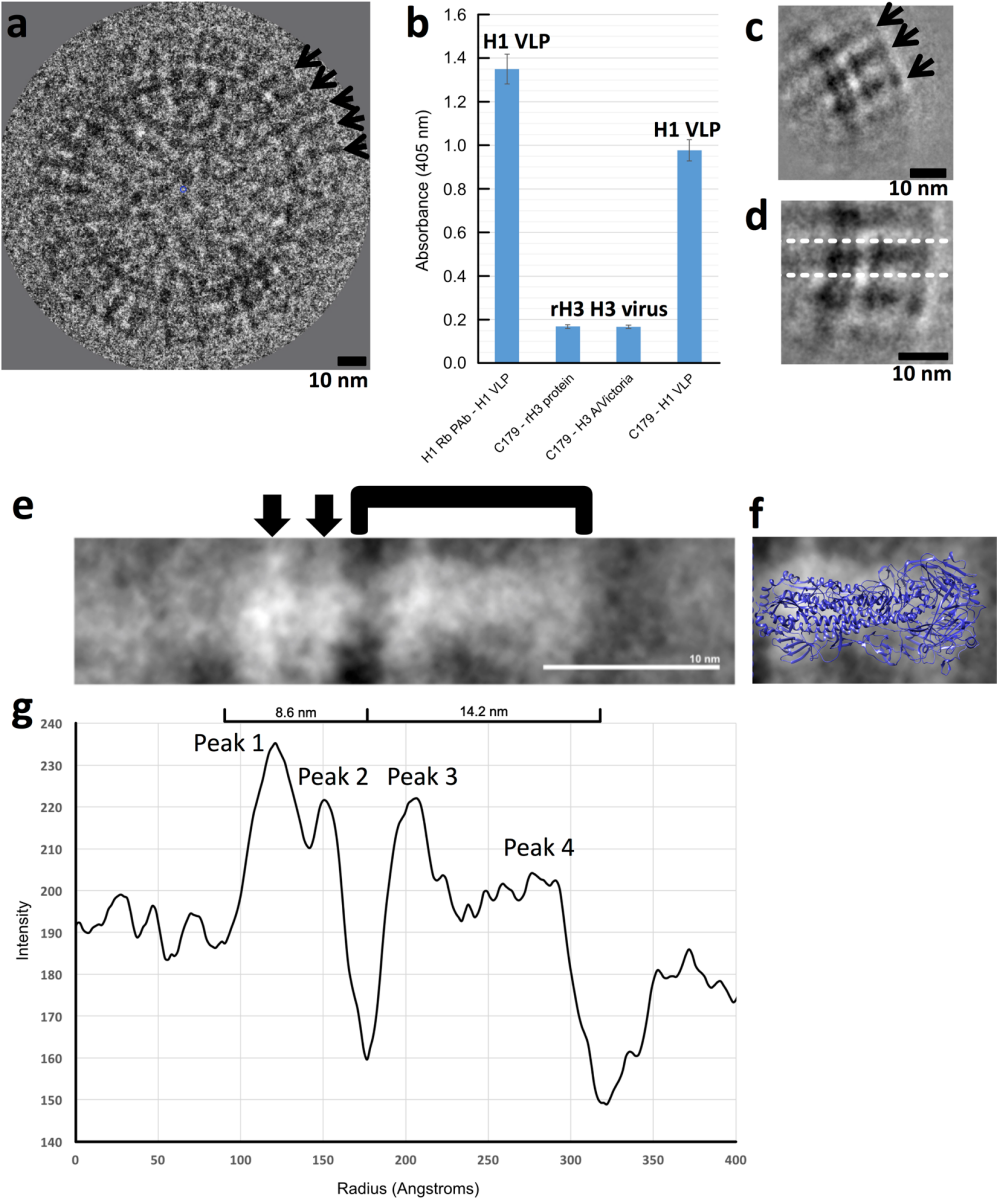
Hemagglutinin (HA) conformation and epitope integrity on virus-like particles analyzed by 2D image analysis and reactivity to antibodies. (**a**) Image of a VLP by cryo-electron microscopy. Black arrows indicate some HA spikes on the surface. (**b**) ELISA of binding of anti-H1 rabbit polyclonal antibodies and stem antibody C179 to VLPs. H3 virus (A/Victoria/3/75 H3N2) and recombinant H3 HA (A/Wisconsin/67/05 (H3N2)) were negative controls because C179 is a broadly reactive antibody for group 1 HAs (e.g. H1, H2, H5) and does not bind group 2 HAs (e.g. H3). (**c**) 2D class average of spike regions from multiple VLP surfaces. Black arrows indicate HA spikes. (**d**) Image of panel c computationally rotated so the central HA spike (within dotted the lines) is horizontal. For panels a, c, and d protein contrast is shown as black. (**e**) Zoomed in view of the 2D HA averaged image. Black arrows indicate two apparent density layers. The HA ectodomain region is marked by a bracket. (**f**) Size and shape comparisons between the ectodomain region of the HA 2D image average and the HA crystal structure (PDBID 1RD8) by scaled image overlay. HA coordinates are in blue ribbons. Protein contrast is shown as white for the 2D average in panels e and f. Panels e and f are on the same scale. All scale bars, 10 nm. (**g**) 1D density profile of the HA average. The relative widths of two large major bands (8.6 nm and 14.2 nm) are denoted by labeled distance brackets that span minima. The 14.2 nm band has multiple peaks while the 8.6 nm band has two peaks.

HA spikes were observed on the surfaces of VLPs (Figs 3a, S6). In order to address the questions of HA conformation and epitope display of HA on VLPs, 2D-image analysis of cryo-EM images was performed along with immunoassays with antibodies to probe for epitope integrity and accessibility. By ELISA HA VLPs bound polyclonal antibodies and the antibody C179 that binds the conserved stem region and requires the prefusion state of HA (Fig. 3b). The VLPs were also reactive to cross-reactive H1 antibodies via western blotting (Fig. S7). However, in order to probe the global conformation of HA molecules from the VLP population we used image averaging in which hundreds of HA molecules from different VLPs were averaged and this average analyzed. Image analysis of the HA average indicated a bi-lobed structure embedded in a bilayer, which was consistent with HA being in a prefusion state (Fig. 3c–g). Furthermore, additional 1D profile image analysis of a spherical VLP indicated a bi-lobed structure for HA on the VLP surface (Fig. S6). The image contrast and observation of the HA spike was enhanced by 2D image averaging. This was done by computationally picking, aligning and averaging patches of HA spikes from the surfaces of VLPs (Fig. 3a). In the average image patch, a central HA was surrounded by neighboring HA spikes (Fig. 3c, arrows). The density distribution in the central HA was further analyzed by image rotation to make the central HA lie horizontally (Fig. 3d, white lines). A zoom-in view of the HA average presented a peanut-shaped profile with two density layers underneath (Fig. 3e, bracket, arrows, respectively). There was shape complementary when the 2D HA image average was compared to the coordinates of the trimeric HA ectodomain via overlay of the two images on the same scale (Fig. 3f). 1D profile analysis of the HA average indicated an ectodomain of about 14.2 nm in size then two density bands spanning about 8.6 nm based on distances between minima (Fig. 3g). However, within the large 8.6 nm width peak there are two peaks that are about 3.0 nm apart (Fig. 3g) corresponding to the separation of the two density bands (membrane bilayer) under the HA ectodomain (Fig. 3e, arrows). We further studied the structure of HA on VLPs by cryo-electron tomography.

### 3D analysis of HA conformation and surface distribution

**Figure 4:**
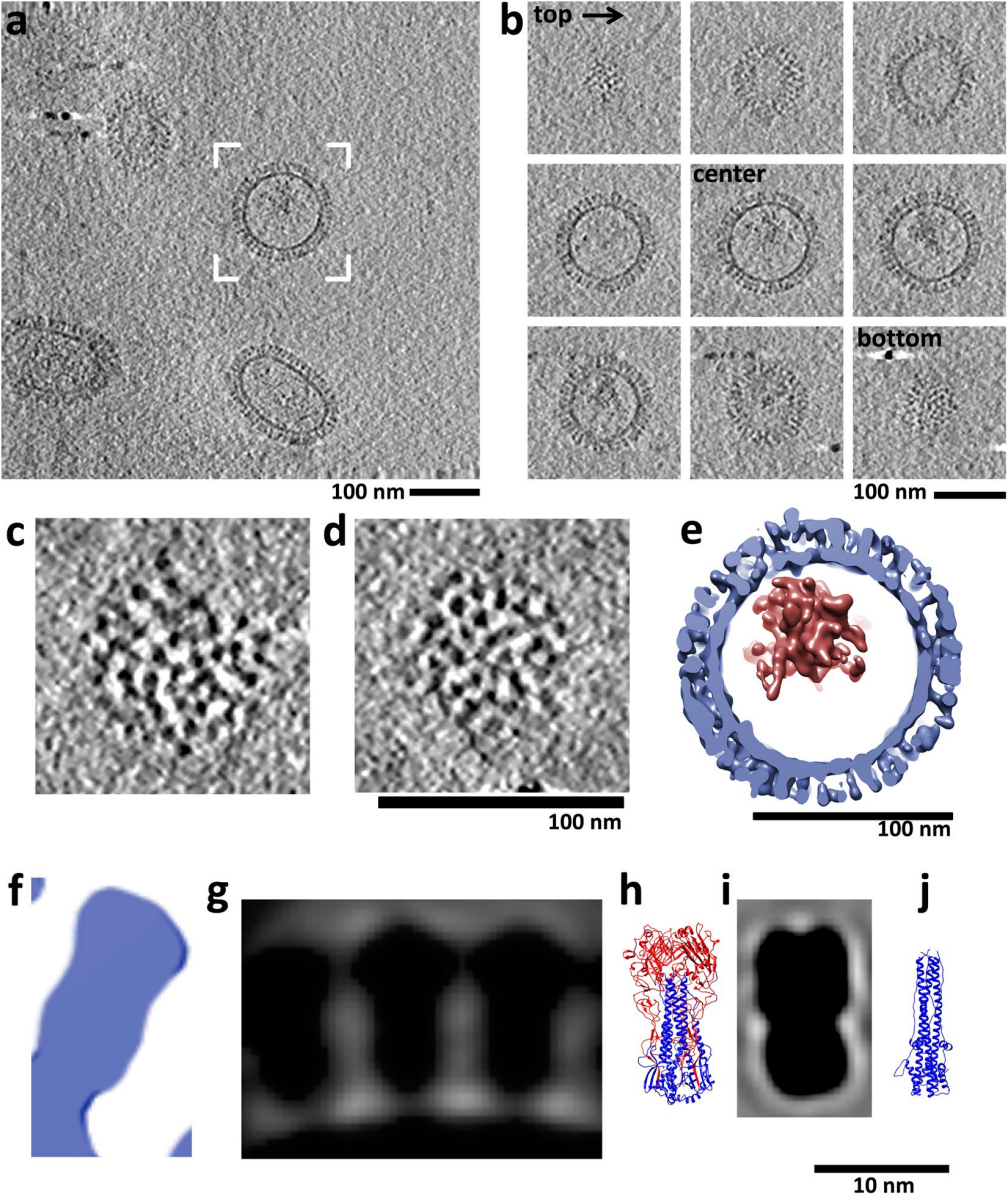
3D Analysis of hemagglutinin (HA) surface spikes of VLPs by cryo-electron tomography. (**a**) Near-central slice through a tomogram of a field of VLPs. (**b**) 2D image slices as iterative z heights from the top through the center and to the bottom surface from the bracketed VLP in panel a. (**c**,**d**) Near tangential slices through two VLP tomograms to indicate near apical (top) views of HA spikes. Protein contrast is black. Panels c and d are on the same scale. (**e**) Structurally segmented model of the VLP in panel b shown as an isosurface rendering. Hemagglutinin and membrane layers are colored light blue with internal components colored brick red. Scale bars (**a**–**e**), 100 nm. (**f**) A slice through a rendered surface of an HA molecule from the VLP shown in panel b. (**g**) Near-central slice through a subtomogram average of HA spikes from VLP surfaces. (**h**) Ectodomain coordinates for H1 HA from 1918 (PDB ID 1RD8) with HA1 and HA2 regions in red and blue, respectively. Coordinates represent a prefusion model of HA. (**i**) Computational simulated shape model from the HA ectodomain coordinates. (**j**) For structural comparison coordinates (PDB ID 1QU1) for a postfusion model of HA is shown within HA2 in blue. Panels f to j are on the same scale. Scale bar, 10 nm.

To understand the 3D conformation of HA and how HA molecules were arranged in 3D over the surfaces of VLPs, cryo-electron tomography was used to obtain 3D structures of particles. Tomograms (3D volumes) of particles were examined by slicing through the volumes (e.g. Fig. 4a–d) and by computational segmentation of particles and HA molecules (Fig. 4e,f). 3D averages of HA molecules from VLP surfaces where derived and then compared to previously reported prefusion and postfusion ectodomain coordinates from x-ray crystallography (Fig. 4g–j). Within near-central slices of VLP tomograms approximate lateral (side) views of HA spikes were attached to membranes that appeared as dark circles and ovals at the base of the HA spikes (Fig. 4a). HA molecules were over the entire surfaces of the particles as revealed by slicing from the top, through the center, and to the bottom of VLPs (Fig. 4b). Slices tangential to the top and bottom of VLPs displayed dot-shaped densities of HA indicative of relative top or apical views of constituent HA molecules (Fig. 4b, top and bottom; Fig. 4c,d). HA molecules were not organized in an apparent symmetrical array on the surfaces but there were no bald areas devoid of HA molecules. Neighboring HA molecules appeared in close proximity giving a rather dense corona of HA spikes on the surfaces (Fig. 4c,d). In order to determine hemagglutinin (HA) spacing, the mean distance between 153 HA pairs was calculated from apical tangent z-slices of VLP tomograms. The average HA to HA distance was 8.2 nm, with a standard deviation of 1.9 nm. Near central slices displayed side views of HA molecules with spike appearances (Fig. 4e,f). On further examination of the spike region, the slices had bi-lobed shaped structures that could also be described as peanut-shaped (Fig. 4f). This shape was similar in contour to the crystal structure of the HA ectodomain in the prefusion state (Fig. 4h) and not like the thinner post-fusion crystal structure of HA (Fig. 4J). In order to further analyze the 3D conformational state of HA on VLPs we carried out subtomogram averaging. 3D spikes volumes (Fig. 4e) on the surfaces of VLPs were computationally extracted and averaged. The subtomogram average indicated bi-lobed, peanut shaped densities (Fig. 4g) that were next to each other on a membrane surface (Fig. 4g). The HA 3D shapes are consistent with the prefusion state of HA (Fig. 4h). When the coordinates of the HA ectodomain (Fig. 4h) are computationally filtered it produced a shape model (Fig. 4i) similar to the subtomogram averaged HA spikes from the VLP cryo-tomography data (Fig. 4g vs. i). Thus, both 2D and 3D structural analysis of HA on VLP surfaces are consistent with HA molecules maintaining a prefusion conformation which would be important in maintaining and displaying conserved stem epitope integrity.

### Internal component of VLPs

Interestingly, tomography and subsequent computational segmentation of a VLP indicated multiple structural components (Fig. 4e). Tomography allowed the internal components of VLPs to be visualized and analyzed. There was an HA-membrane layer (Fig. 4e, blue) and observed density inside corresponding to internal components (Fig. 4e, red, Fig. S8). The internal component was not a solid regular density but had an irregular shape with spaces between the local density masses (Fig. S8).

**Figure 5:**
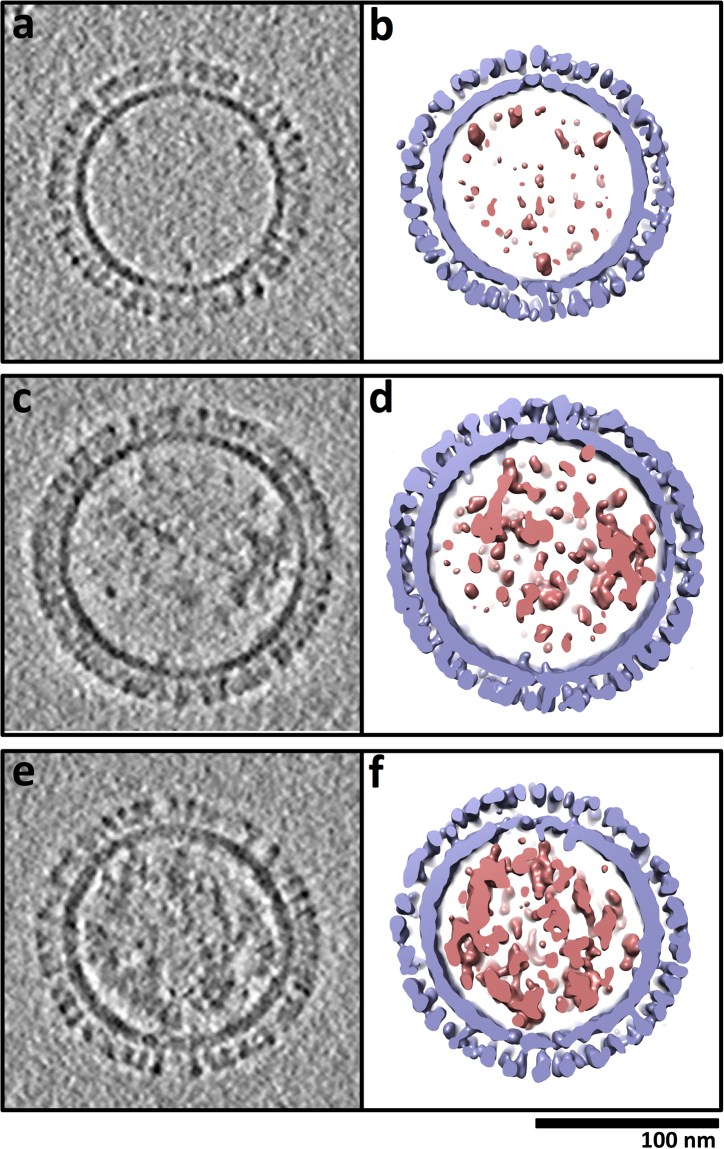
Comparison of the relative amounts of internal components between virus-like particles (VLPs). (**a**,**c**,**e**) Near-central slices through VLPs judged by comparison to have low (panels a, b) medium (panels c, d) and large (panels e, f) amounts of material inside the particles. Panels a, c, and e have the VLPs displayed as grey-scale images and panels b, d, and f correspond to the VLPs as isosurface renderings. In the segmented isosurface renderings (**b**,**d**,**f**) hemagglutinin and membrane are light blue and internal components (molecular cargo) are colored brick red. Panels are on the same scale. Scale bar 100 nm.

In order to further understand the 3D organization of the internal components of the VLPs, hundreds of tomograms of individual VLPs and internal components were analyzed for organization and the amount of internal component. Select examples were displayed as a montage (Fig. 5). All tomograms (3D volumes) of VLPs (N = 353) analyzed contained observable internal components (Fig. 5, red). Although VLPs had dense layers of HA spikes on their surfaces (Fig. 5, light-blue), they did vary in the amount and distribution of their internal components (Fig. 5, red). The irregular organization of the internal components was a consistent characteristic of hundreds of individual tomograms of 3D structures of VLPs that were examined.

**Figure 6:**
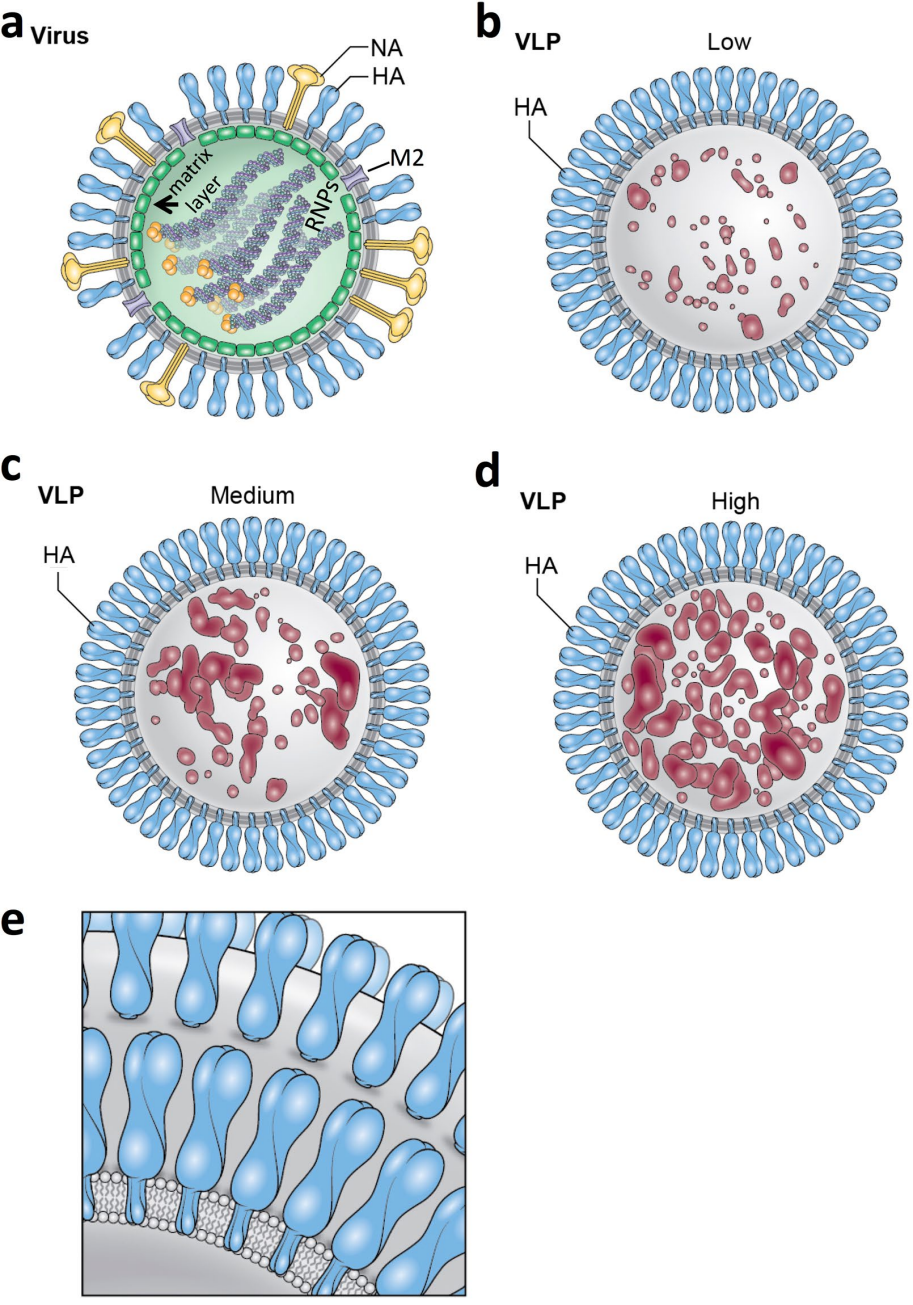
Structural schematics for the comparison of the molecular organization of influenza virus and virus-like particles (VLPs). (**a**) Schematic of an influenza virus particle. (**b**,**c**,**d**) Schematics of VLPs containing low, medium, and high amounts of internal components, respectively. Hemagglutinin (HA) is light blue with the membrane in grey. Internal components of VLPs are brick red. For the virus, additional components are viral glycoproteins, which are neuraminidase (NA) (yellow), M2 (light purple), matrix layer (green) with genomic RNP filaments inside with polymerase proteins (yellow) at one end. For clarity only one row of surface glycoproteins is shown. (**e**) Schematic of HA on the surface of VLPs illustrating neighboring HA molecules that are besides, in front, and behind each other on the surface.

When compared to vesicles, which was less than one percent of the particles, VLPs had HA spikes on the surfaces and internal components (Fig. S9a vs. b). Also, the internal components were not arranged as filaments like the genomic RNPs in tomograms of influenza virus particles (Fig. S2d–i). The VLPs lacked an apparent matrix layer (Fig. 5) like influenza viruses (Fig. S2). The apparent amounts of encapsulated internal components varied among VLPs and this was used to designate VLPs into three types (low, medium, high). This was based on the apparent visual amount of internal contents compared to unoccupied space inside the VLPs (Fig. 5) (Movies S1, S2, S3). Low was defined at less than 25% filled, medium as 25–50% filled and high as greater than 50% filled. The population of VLPs consisted of about 26% low-filled, 43% medium-filled, and 31% high-filled (Fig. 5) and this could be depicted schematically (Fig. 6). In supplemental analyses (Fig. S10, Supplemental Methods) semi-automated methods were used to determine the relative amount of internal density (%fill) for VLPs and it was found that all VLPs had internal densities with the majority trending toward spherical morphology (Fig. S10). No reoccurring patterns within the internal component regions of VLPs were observed (Fig. 5).

## Discussion

In order to answer questions about the composition and morphology of influenza virus-like particles (VLPs) and the organization and conformation of constituent hemagglutinin (HA) molecules, we studied VLPs made with the H1 HA from the 1918 influenza pandemic virus. VLPs were studied by biochemical analysis along with structural studies by cryo-electron microcopy and tomography coupled to image analysis. Our results indicated size variation among the VLPs but with most VLPs being ascribed to a spherical morphology. HA was the major component of the VLPs and hundreds of HA molecules were displayed on VLP surfaces with HA in an apparent prefusion state. Despite having an almost undetectable amount of the internal matrix (M1) protein, the VLPs did contain internal component densities. Inclusion of internal components (i.e. molecular cargo) was observed in all VLPs. We postulate based on mass spectrometry (Table S1) that internal components could consist of encapsulated cellular components that facilitate VLP assembly.

### Cellular proteins of influenza viruses and virus-like particles

Understanding influenza virus-like particle (VLP) composition maybe important because compositional studies of influenza virus particles from different cells identified cellular factors that may function in influenza virus assembly, entry, and replication^15–17^. In previous studies purified influenza virus particles were subjected to proteomic analyses using mass spectrometry to identify cellular proteins^15–17^. Analyses of viruses from mammalian and avian cells identified host proteins in categories such as cytoskeletal proteins, annexins, glycolic enzymes, and tetraspanins^16^. Other studies of virus-host protein interaction networks (i.e. interactome) for influenza virus identified cellular factors such as plakophilin 2 (cytoskeletal-associated protein) that could restrict influenza replication^17^. For our study, SDS-PAGE, immunoassays, cryo-electron microscopy and peptide-finger printing identified influenza H1 HA as the major component of 1918 influenza VLPs (Fig. 1). However, what might be the constituents of a VLP dominated in composition by HA and from a baculovirus expression system had not been addressed. Previous studies indicated that influenza viruses and VLPs that contained other influenza structural proteins such as NA, M1, M2, and NP could bind and interact with mammalian and avian cellular proteins during virus infection and VLP formation^13,15,16,18–21^.

In this work, the highest frequency of peptide matches (n = 173 peptide matches) were to HA, while the next highest matches of peptides were to cellular proteins of Sf9 cells: heat shock cognate 70 (n = 6), heat shock protein 70 A1 (n = 4), actin (n = 4) peptide matches. Only one peptide (n = 1) matched to the matrix protein M1 and only one peptide (n = 1) to an open reading frame from baculovirus (Table S1). Matrix protein M1 was deemed to be in low amounts because only one peptide of M1 was detected and no full-length M1 protein was biochemically detected and no M1 layer was observed in VLPs by cryo-electron microscopy. One plausible reason for the low amounts of M1 could be that the M1 expressing baculovirus was less effective than the HA expressing baculovirus. VLPs were made by co-infection of Sf9 insect cells with two separate baculoviruses with one expressing HA and the other expressing M1^14^. Based on these results, we suggest that some cellular proteins of Sf9 cells could be involved in influenza VLP assembly, but HA remains the dominant component of the VLPs (Fig. 1a). Whether baculovirus macromolecules can play a role in influenza VLP assembly in this system is unknown since we detected no baculovirus particles in our cryo-EM images (Fig. 1d) and only one baculovirus peptide was detected at low frequency (Table S1). One notion is that HA was the dominant component on the surface of the VLPs and cellular proteins act as encapsulated cargo. This implies a HA-dominated assembly mechanism where baculovirus glycoproteins could be excluded by high HA protein levels. It will be interesting to study other influenza VLP systems in the future to determine similarities and differences in composition.

### Composition and morphology relationships

Our structural analyses of VLPs allow for comparing and contrasting morphologies and compositions of VLPs with influenza virus particles. Influenza proteins NA, M2, and matrix (M1) have been implicated in virus assembly, budding, and morphology^19,22–29^. Furthermore, the dominant method for VLP production has been the co-expression of matrix M1 with one or all viral glycoproteins, HA, NA and M2. Influenza viruses can have spherical and filamentous morphologies^11,12^ (Figs S2, S4). Interestingly, previous studies using reverse-genetics to make mutant viruses indicated that changing the matrix protein can alter the proportion of spherical to filamentous virus morphologies^22,30–32^. However, the matrix protein of influenza virus is a major structural protein and is required for influenza budding and genomic RNP incorporation into virions during infection^18–20,30,33,34^. Thus, deletion of the matrix gene is not feasible for the production of mutant viruses.

In terms of composition, our biochemical analysis of 1918 H1 HA VLPs indicated that HA was the major component and constituted over 90% of the protein (Figs 1a, S1). This is different from influenza viruses in which HA is less than half of the total protein component with more being contributed by structural proteins NP and matrix M1 (Fig. S2b,c). In terms of morphology, it has been generally established that influenza virions vary in size and are pleiomorphic with spherical and filamentous morphologies^6,11–13^. In this study, we present supplemental work of imaging influenza viruses to demonstrate the spherical and filamentous natures of influenza viruses under similar imaging conditions as VLPs (Figs S2, S4). However, the size variation and 3D morphologies and structures of influenza VLPs used as vaccine immunogens had not be addressed in great detail^14,21^. Our observed size variations using hundreds of VLPs studied by cryo-EM (Fig. 2) were similar to that observed for influenza virions^6,11–13^. The average VLP length based on a membrane boundary was 87.3 nm and ranged from 29.7 nm to 221.5 nm. Thus, the calculated average diameter of a VLP would be 119.7 nm. This takes into account the glycoprotein layer size (14.2 nm ectodomain and 2 nm stem, Figs 3e–g, 4f–h) added to the average size (87.3 + 14.2 + 14.2 + 2.0 + 2.0 nm). This is slightly smaller but close to the reported average diameters for spherical virions of H1N1 and H3N2 viruses (130 nm, 120 nm), respectively^6,12^. However, VLPs did have diameters up to 221.5 nm (Fig. 2). Thus, these VLPs appear to encompass the morphologies and sizes observed for influenza viruses.

However, very long filamentous VLPs were not observed like for influenza viruses in which filamentous particles can be microns in length (Fig. S4a,b). Thus, these VLPs appeared to be more akin to egg-grown viruses in terms of having more spherical morphologies as opposed to MDCK-grown viruses, which tend to be more populated with filamentous virions. This would make sense if a matrix layer under of the membrane is required to make the long filamentous morphologies. Evidence for this is that mutations in the matrix can change the relative amounts of spherical and filamentous morphologies of influenza viruses^22,30–32^. Of note is that only a small number of the VLPs had appearances that could be described as short filaments (Fig. 2f). This is in agreement with our reasoning that without matrix levels similar to viruses that the dominant morphology of HA VLPs is predominantly spherical. This perhaps is dictated by the glycoprotein HA in the absence of other viral proteins (e.g. matrix (M1), nucleoprotein (NP)) levels found in the virus (Figs 1 versus S2). Whether this is specific or not to H1 HA for the 1918 influenza pandemic virus can be addressed in future studies based on the work presented in this study of VLPs.

### Lack of internal viral structural proteins of VLP systems

The methodology that is currently most used to make influenza VLPs is over expression of viral glycoproteins, HA, NA, M2, and the internal structural protein, matrix protein M1. However, we identified VLPs similar to influenza viruses in morphology, but lacking a discernable matrix layer and content like viruses, but with internal cargo that we propose could act as a matrix surrogate. Additional evidence for the use of a surrogate for the influenza matrix comes from work with HA VLPs that used retroviral GAG polyprotein as the internal major structural protein instead of influenza matrix protein^35,36^. Furthermore, other viral VLPs can form glycoprotein particles without their internal capsid structural proteins. For example, the glycoproteins of hepatitis B virus can form particles without the internal structural capsid protein^37^. Likewise, glycoproteins of Zika virus can form particles without expression of the capsid protein^38^. Additionally, recombinant hemagglutinin proteins without matrix and a membrane can form complexes^39^ but they are smaller in size and HA numbers than influenza viruses and VLPs studied in this work. Together, results from previous work on viral glycoprotein particles and complexes used as vaccine immunogens and observations from our 1918 H1 HA VLPs suggest that both capsid and matrix structural proteins maybe dispensable for virus-like particle (VLP) and nanoparticle production. On rare occasions, smaller VLPs were observed inside of larger ones by cryo-electron microcopy (Fig. S9c). Although we cannot rule out that HA and cellular components might have displaced matrix during particle formation, we speculate that the internal structural component that we observed inside of VLPs could be composed of cellular components that somehow were encapsulated inside VLPs (Figs 5, 6). The possible importance of the size of this internal density distribution and possible roles in vaccine development could be addressed in the future through structure-guided design efforts of VLPs in order to engineer distinct distributions.

The 3D structural observations on apparent plasticity of content packaging and arrangement of influenza vaccine VLPs as described in this work may have bearing on understanding some previously reported influenza VLP biotechnology engineering work that did not present detailed 3D cryo-EM analysis. For example, cellular proteins have previously been engineered to be assembled into influenza VLPs and other non-influenza viral epitopes have been fused to VLP glycoproteins^40–42^. In one study, influenza VLPs with GPI-anchored CCL28 (mucosae-associated epithelial chemokine, MEC) induced long-lasting mucosal immunity against H3N2 viruses. This was termed as a mucosal adjuvant^40,41^. Thus, our observation of influenza VLPs with proposed internal cellular components would be on a continuum of future studies that maybe important in further engineering influenza VLPs with improved immunogenicity. This could be done by encapsulating conserved influenza epitopes or cellular proteins that can boost the immune responses by engineering in membrane-targeting signals such as GPI-anchors onto proteins. Such structure-guided design of novel influenza VLPs could lead to more efficacious influenza vaccines that protect against different strains and subtypes and aid universal influenza vaccines efforts^14,43–47^.

### Conformational state of hemagglutinin (HA)

Many viral glycoproteins, such as influenza HA, undergo conformational changes that mediate membrane fusion during viral entry and uncoating^48^. In the case of influenza, some anti-HA antibodies can block fusion activity by binding to the prefusion state of HA and inhibiting the conformational changes needed to mediate fusion. Hence, there is great interest in displaying influenza HA and other viral glycoproteins in a prefusion state on vaccine immunogens such as membrane-containing VLPs^14,43–47,49^ and non-membrane containing nanoparticle complexes^7–9,39,50–52^. Thus, a number of vaccine efforts have focused on the development of more efficacious influenza vaccines that would elicit antibodies to the more conserved stem region of HA that is involved in mediating fusion^5,52–55^. One reported vaccine strategy to elicit broadly neutralizing antibodies to conserved HA epitopes in the prefusion state was to engineer HA proteins with the stem region only in that the HA head region was removed. Also, in another method chimeric HA proteins with antigenically different head regions but with conserved stem regions were used to boost stem antibodies^46,52,56,57^. Concerning the 1918 VLPs in this study, they were shown to be efficacious in animal models^14,58^, but the assessment of reactivity with stem antibodies and structural conformational state of HA within the VLPs were in not explored in detail. In this study, we showed by cryo-electron microscopy that HA on 1918 VLPs were consistent with a prefusion state (Figs 3, 4) and HA could bind cross-reactive polyclonal antibodies and stem antibodies (Figs 3b, S7). These results imply that a matrix layer under the membrane and other viral glycoproteins such as NA and M2 are not required to maintain the prefusion state in which conserved stem epitopes are displayed.

By cryo-electron microscopy the 1918 VLPs had of corona of surface spikes similar to H1N1 viruses (Figs S2, S4). Viruses were limited to APR8 H1N1 and CA09 H1N1 viruses which are biosafety level 2 viruses. It is logistically challenging to study biosafety level 3 or 4 infectious viruses with infrastructure required for cryo-electron microscopy. In the context of shedding light on the potential use of VLPs as vaccine platforms it will be informative to use the same virus strain with and without the RNPs (infectious versus noninfectious) to understand specific differences and similarities as well as across virus strains. Although not ideal because the native structure might be perturbed, one way to study biosafety-level 3 influenza strains is to chemically fix them prior to imaging via cryo-EM^59^. This is something that could be done in future studies with 1918 influenza viruses for structural comparisons to influenza VLPs and other influenza viruses.

In conclusion, our results from this study of VLPs of the 1918 pandemic influenza virus suggest that for these VLPs that HA is the major constituent. HA was observed as spikes on the surfaces of VLPs. Multiple internal molecular components were observed and attributed to some possible cellular components based on proteomic analyses. We term the internal components as molecular cargo. This suggests that the underlying mechanism of VLP structural organization for these VLPs is dominated by HA and possibly aided by the internal molecular cargo (Fig. 6). VLPs varied in sizes with a mostly spherical particle morphology in the population. HA on the surface was in a prefusion state and conserved stem epitopes were displayed for antibody binding. This suggests that HA folding and assembly into VLPs did not trigger the pre- to post-fusion transition for surface molecules. The analyses presented here for 1918 H1 HA VLPs should prove useful in assessing the molecular organization of VLPs and constituent HA molecules across various influenza hemagglutinin (HA) subtypes. This could aid in the development of more efficacious seasonal vaccines and assist universal influenza vaccine efforts.

## Methods

### Biochemical analysis

Virus-like particles (VLPs) of the 1918 H1 VLP were prepared as previously reported^14^ in which VLPs protected animals from influenza challenge. In brief, hemagglutinin (HA) H1 VLPs were purified from a baculovirus expression system via the co-expression of HA from influenza virus A/South Carolina/1/1918 (H1N1) and matrix protein M1 of influenza virus A/New York/312/2001 (H1N1), respectively^14^. Total protein concentration was determined to be ~0.84 μg/μl based on bicinchoninic acid (BCA) protein assay (ThermoFisher). VLP purity and protein composition along with HA cleavage status were analyzed biochemically by methods such SDS-PAGE and subsequent peptide fingerprinting by mass spectrometry. Samples were analyzed under reducing conditions with the reducing agent dithiothreitol (DTT) at a final concentration of 100 mM and heated at 95 degrees Celsius for 10 minutes. Gels were stained over-night with coomassie blue stain (SimplyBlue SafeStain, Invitrogen) for protein band visualization and then subsequently subject to densitometry analysis. Gels were scanned and digitized into images with a gel documentation system (Enduro GDS). One-dimensional profiles of the gel lanes for VLP and molecular weight standards were calculated and areas under profile peaks were analyzed with the image processing software, Fiji^60^.

For peptide finger printing the major band at ~75 KDa of the VLP sample was excised from the gel and subjected to protease digestion to create tryptic peptides that were analyzed by mass spectrometry (MS). Acquired MS/MS spectra were searched against and mapped on the expected 1918 H1 HA sequence. Spectra were searched against three constructed proteome databases: (i) influenza virus, (ii) Sf9 cells, and (iii) baculovirus using the program SEQUEST and Fixed Value Peptide Spectrum Match (PSM) validator algorithms in the Proteome Discoverer 1.4 software (Thermo Scientific, CA)^61^. The total numbers of PSMs from searches were 174 from the influenza database, 23 from the Sf9 cell database, and 1 from the baculovirus database. Within the influenza virus database search 99.4% of the PSM were to hemagglutinin and the highest match was 108 PSMs to an influenza hemagglutinin H1: Accession (Q9WFX3), Description (Hemagglutinin OS = Influenza A virus (strain A/Brevig Mission/1/1918 H1N1) GN = HA PE = 1 SV = 2 − [HEMA_I18A0]. Sequences were from Gene Bank: AF117241.1 for HA H1 of Influenza A virus (A/South Carolina/1/1918 (H1N1)); Q9WFX3.2 for HA H1 of influenza A virus (A/Brevig Mission/1/1918 (H1N1)). Sequence alignment between the H1 HA sequences of H1 of influenza A/South Carolina/1/1918 (H1N1) and H1 of A/Brevig Mission/1/1918 (H1N1) was done with the program EMBOSS-Lite with the sequences being 100% identical. Identified peptides from mass spectrometry was mapped onto the H1 HA (1918) ectodomain crystal structure from the Protein Data Bank (PDBID 1RD8)^62^ using Chimera software package^63^. Further details on the concepts of Peptide Spectrum Match (PSM) and coverage in mass spectrometry can be found in the user guide for the Proteome Discoverer software (https://tools.thermofisher.com/content/sfs/manuals/Man-XCALI-97506-Proteome-Discoverer-14-User-ManXCALI97506-A-EN.pdf).

### Immunoblotting

Reactivity of H1 HA of VLPs with antibodies was assessed by western blotting and ELISA. Purified rabbit polyclonal antibody raised against purified recombinant H1 HA of influenza A/California/07/2009 (Protein Sciences Corporation, Meriden, CT) was used as primary antibody to probe for reactivity to H1 HA 1918 VLPs by Western blot analysis using standard procedures. Blots were probed with secondary goat anti-rabbit HRP and subsequently visualized using SuperSignal West Pico chemiluminescent substrate (Thermo Fisher Scientific, Waltham, MA) to expose film. The H1 rabbit polyclonal is cross-reactive to different HA H1s, but specific for H1 subtype and thus recombinant H3 HA (A/Wisconsin/67/05 (H3N2) and recombinant H1 HA (A/PR/8 (H1N1) proteins were used as positive and negative HA subtype controls, respectively. Thus, HA H3 is an influenza HA from group 2 influenza virus (negative control) and H1 from APR8 was a cross-reactive H1 (group 1, positive control). All recombinant HA proteins were full-length uncleaved HA0 and purified from baculovirus expression systems (Protein Sciences Corporation).

### ELISA

Reactivity of 1918 H1 HA of VLPs to a stem antibody that requires a conformation epitope was probed by ELISA using the monoclonal antibody C179, which has been shown to bind to the conserved stem region of HA H1 2009 pandemic virus^6^. Antibody C179 was from Takara Bio USA. In addition, 1918 H1 HA VLPs were probed via ELISA with the pan H1 rabbit polyclonal that had shown reactivity in western blot. ELISA plates were coated with samples at a concentration of 2 μg/ml, before incubating with C179 antibody or rabbit pan H1 polyclonal antibody (1.5 ug/ml). Samples were 1918 H1 HA VLPs and H3 virus (A/Victoria/3/75 H3N2) and recombinant H3 HA (A/Wisconsin/67/05 (H3N2)) protein as negative controls because C179 does not react with H3 HAs. Subsequent detection was with a horseradish peroxidase conjugated to goat anti-mouse IgG and goat anti-rabbit IgG for detection. Binding was quantified by the addition of ABTS substrate (Thermo Fisher Scientific, Waltham, MA) and optical density was measured at a wavelength of 405 nm. Samples were in triplicate.

### Electron microscopy

Negative-staining electron microscopy of VLPs was similar to that reported previously for other samples^37,64^, except that VLPs were stained with 1.5% phosphotungtic acid. Images were collected on a Tecnai 12 electron microscopy with LaB6 illumination operating at 100 kV (FEI, Eindoven, Netherlands) at nominal magnifications of 52,000x and 110,000x. Images were recorded on a 4k × 4k OneView camera (Gatan, Pleasaton, CA). For cryo-electron microcopy, 3.5 μl of unstained VLPs were applied to glow discharged 200 mesh R2/2 Quantifoil Cu grids (Quantifoil, Großlöbichau, Germany) and plunge frozen using a Vitrobot Mark IV plunger (FEI Company, Hillsboro, OR). Samples were imaged under cryo conditions at 300 kV on a Titan Krios electron microscope (FEI, Hillsboro, OR). Images were collected using EPU software (FEI Company, Hillsboro, OR) on a 4k × 4k Gatan Ultrascan charge-coupled-device (CCD) camera (Gatan Inc., Pleasanton, CA) 1.9 Å pixel size (nominal magnification 47,000x). Images were collected with a defocus range of −3 to −5 μm and the electron dose ranged from ~10 to 20 e^−^/Å^2^. Individual VLPs from 2D cryo images were further analyzed by 2D image analysis.

### Cryo-electron tomography

VLP samples were mixed with 5 or 10 nm gold particles as fiducials and plunge-frozen as above. Tilt series were collected using a GIF-2002 energy filter (slit width, 20 eV) coupled with a 2k × 2k MultiScan Gatan Ultra CCD (Gatan Inc., Pleasanton, CA). For lower defocus data, tilt series were collected using a Falcon II direct-detector camera using a Volta phase plate (VPP) (FEI, Hillsboro, OR). Zero loss energy filtered data was collected with a 70 μm objective aperture at 3.3 Å pixel size (nominal magnification 26,000x) with a defocus range of −3.5 to −5.5 μm. Tilt series collected with VPP on Falcon II was acquired at 2.9 Å pixel size (nominal magnification 29,000x) with a −2.5 μm defocus. All single-axis tilt series data were collected over −/+ 60 degrees with 2° tilt increments using Tomography 4.0 software (FEI, Hillsboro, OR). Tilt series datasets were collected under low-dose conditions with a cumulative electron dose of ~100 electrons/Å^2^. Images within the tilt series were mutually aligned by using the gold particles as fiducial markers and the 3D volumes (tomograms) were reconstructed as implemented the IMOD software package^65^.

### Size and morphology measurements

Size parameters such lengths and shape morphology were measured from 2D cryo-EM images of VLPs (N = 295). The dark membrane perimeters appearing in VLP images were modeled as ellipses. Ellipses with varying major and minor axes were semi-automatically fit to VLP membrane perimeters using the image processing software, Fiji^60^. The major axis and minor axis of the fit ellipsoid were recorded. Morphology was assessed by the VLP aspect ratio, which was the major (longer) axis divided by the minor (shorter) axis. A histogram of the aspect ratios of the VLP population and a heat map of the aspect ratios vs. major lengths were used to analyze the relation between VLP aspect ratios and major VLP lengths. Histogram and heat-map analyses were with the R Project for Statistical Computing software package. VLP sizes and morphologies from different regions of the heat-map were then visually compared via their 2D cryo-EM images.

### 2D and 3D image analyses of VLPs and surface spikes

2D profile analyses were of VLPs and glycoprotein spikes regions. For VLP profiles, individual spherical VLPs with no neighboring obstructions were picked for profile assessment from 2D cryo-EM images. 1D line profile values were extracted from VLP and HA images using Bsoft^66,67^. For VLP analysis a circular average was computationally created and the minima and maxima of the pixel values were normalized to 0 and 1, respectively. Density values were plotted in a 1D profile curve for the membrane-spike region of the VLP. For spike (hemagglutinin) profile analysis, 75 spike regions were picked and aligned and averaged as implemented in EMAN2^68^. Box sizes were 256 by 256 pixels resulting in particle images having multiple but visually separated HA molecules. The selected particle images were cycled through multiple rounds of alignment and averaging, thereby creating a 2D image average with apparent side-by-side delineated HA molecules. The 2D average was computational rotated so that the middle HA was oriented with its long-axis in a horizontal direction with a resulting 1D density profile of the selected middle HA spike and membrane region. Coordinates of the H1 HA ectodomain crystal structure (PDBID 1RD8)^62^ were overlaid on the HA 2D average from cryo-EM on the same scale in Chimera. Subtomogram averaging of HA surface spikes on VLPs were performed similar to as previously reported for ring structures using the software Relion^64,69^. Coordinates (xyz) for spike centers (684) were manually selected using IMOD and passed to Relion to extract 3D volumes (subtomograms) of HA spikes and to carryout subtomogram averaging. Post-processing as carried out in the Relion software package indicated a resolution of 26.5 angstroms. The 3D subtomogram average was compared to HA ectodomain coordinates (PDBIDs, 1RD8, 1QU1) representing prefusion and postfusion HA structures from the protein data bank^62,70^.

### HA spacing, categorization and 3D segmentation of VLPs

3D volumes (i.e. subtomograms) of individual VLPs were extracted from the tomograms that contained multiples VLPs. A total of 353 VLP subtomograms were analyzed. Spacing between HA spikes were measured using line segments traced on both near tangential and central slices of VLP tomograms as analyzed in IMOD^65^. Line-segments were saved as two points with x, y, z coordinates and distances calculated with an in-house python program script. The student t-test showed that there was not a statistically significant difference between HA distances measured by tangential or central sections of VLPs. Because VLPs tomograms revealed internal components (molecular cargo) inside the VLPs, the VLPs were subsequently categorized according to the density of molecular cargo present. Three categories were devised as being low (<25%), medium (>25% to <50%), and high (>50%) pertaining to the relative amount of higher density components relative to lower density space inside the same VLP. VLP segmentations of spike-membrane regions from internal components were carried out with segmentation features within the Chimera software package^63^. Supplemental semi-automated methods were developed and used to determine the relative amounts of internal densities in VLPs for supplemental analysis that is detailed in supplemental methods.

### Data availability

Datasets are available from the authors upon request.

## Electronic supplementary material

Supplemental material

Movie S1

Movie S2

Movie S3
