# Supplementary material for: Structural analysis of influenza vaccine virus-like particles reveals a multicomponent organization
Source: Sci Rep. 2018 Jul 9;8:10342. doi: 10.1038/s41598-018-28700-7 (PMC6037804; doi:10.1038/s41598-018-28700-7)
Supplement: Supplementary file 1 — Supplemental material [file 41598_2018_28700_MOESM1_ESM.pdf]

SUPPLEMENTAL MATERIAL

Structural analysis of influenza vaccine virus-like particles reveals a multicomponent organization

Dustin M. McCraw[1], John R. Gallagher[1], Udana Torian[1], Mallory L. Myers[1], Michael T. Conlon[1], Neetu M. Gulati[1] and Audray K. Harris [1#]

[1]Laboratory of Infectious Diseases, National Institute of Allergy and Infectious Diseases, National Institutes of Health, 50 South Drive, Room 6351, Bethesda, MD, USA 20892

#To whom correspondence should be addressed; E-mail A.K. H. (harrisau@mail.nih.gov) 301-385-4061

Keywords: influenza virus, virus-like particles, cryo-electron microscopy, structure, tomography, vaccine, assembly

Classification: Biological Sciences, Biophysics and Computational Biology

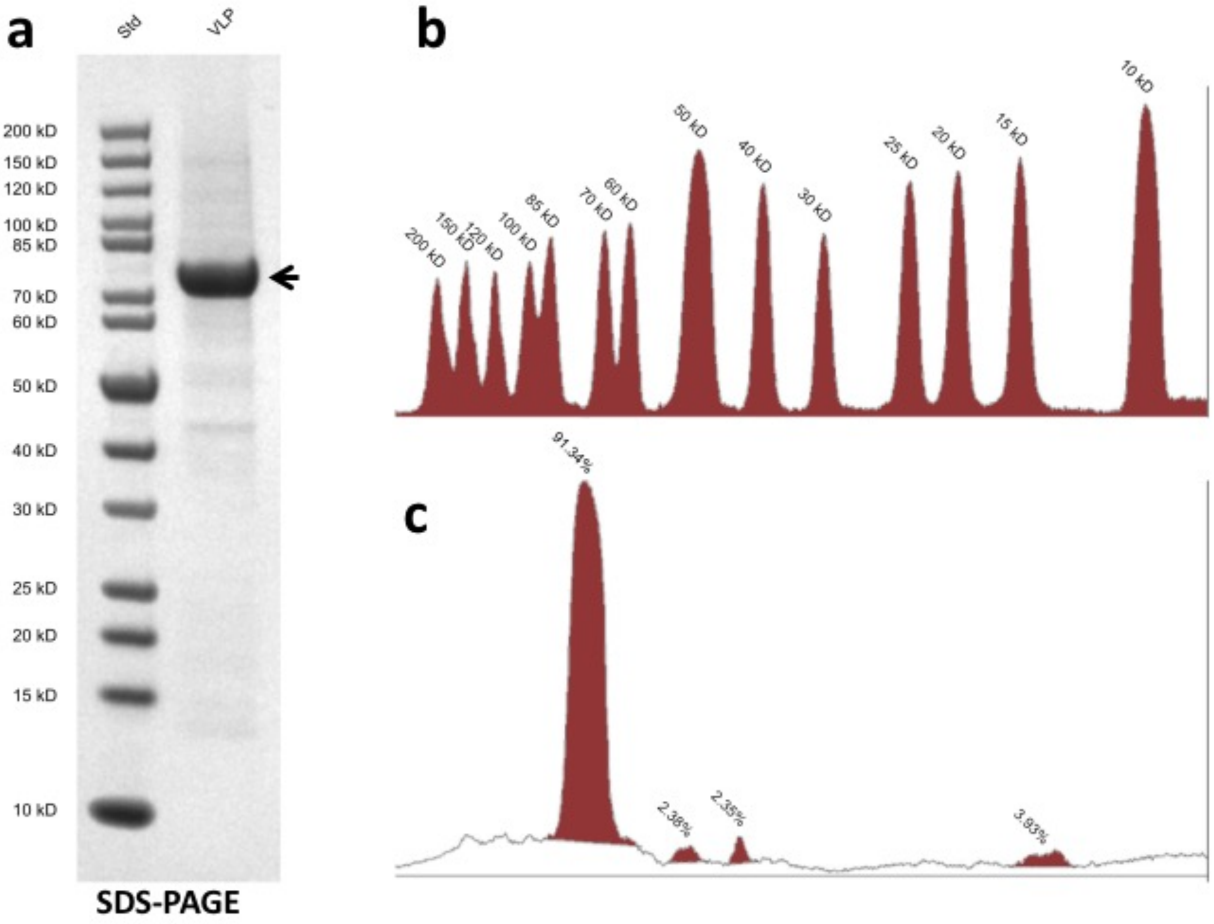
a
Std
VLP
200 kD
150 kD
120 kD
100 kD
85 kD
70 kD
60 kD
50 kD
40 kD
30 kD
25 kD
20 kD
15 kD
10 kD
SDS-PAGE
b
200 kD
150 kD
120 kD
100 kD
85 kD
70 kD
60 kD
50 kD
40 kD
30 kD
25 kD
20 kD
15 kD
10 kD
c
91.34%
2.38%
2.35%
3.93%

Figure S1

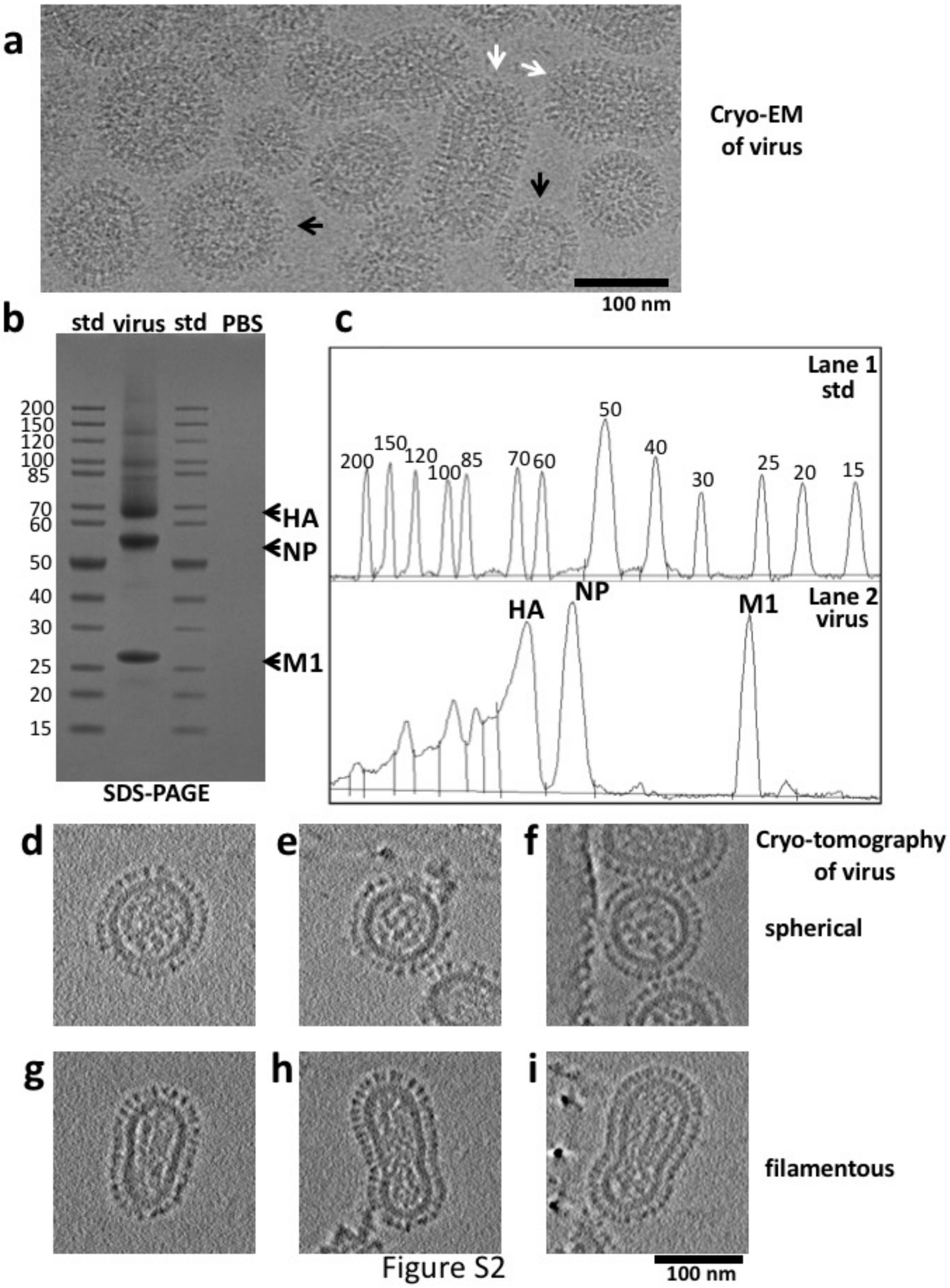
a
Cryo-EM
of virus
100 nm
b
std virus std PBS
200
150
120
100
85
70
60
50
40
30
25
20
15
HA
NP
M1
SDS-PAGE
c
Lane 1
std
200
150
120
100
85
70
60
50
40
30
25
20
15
Lane 2
virus
HA
NP
M1
d
e
f
Cryo-tomography
of virus
spherical
g
h
i
filamentous
100 nm

Figure S2

28

Table S1

TABLE S1. (Peptide Spectrum Matches) of 1918 HA H1 from VLPs)					
**INFLUENZA SERACH**					
**Accession**	**Description**	**# PSMs**	**# AAs**	**MW [kDa]**	**calc. pI**
**P03455**	**Hemagglutinin OS=Influenza A virus (strain A/Swine/New Jersey/11/1976 H1N1) GN=HA PE=3 SV=1 - [HEMA_I76AI]**	**57**	566	63.3	7.56
**Q9WFX3**	**Hemagglutinin OS=Influenza A virus (strain A/Brevig Mission/1/1918 H1N1) GN=HA PE=1 SV=2 - [HEMA_I18A0]**	**108**	566	62.8	6.47
Q82509	Hemagglutinin OS=Influenza A virus (strain A/Tern/South Africa/1961 H5N3) GN=HA PE=2 SV=1 - [HEMA_I61A0]	1	568	64.2	6.23
P87506	Hemagglutinin OS=Influenza A virus (strain A/Mallard/Ohio/556/1987 H5N9) GN=HA PE=2 SV=1 - [HEMA_I87A1]	7	564	63.6	5.92
Q6DPU2	Matrix protein 1 (Fragment) OS=Influenza A virus (strain A/Guinea fowl/Hong Kong/38/2002 H5N1 genotype X0) GN=M PE=3 SV=1 - [M1_I02A1]	1	245	27.1	9.42
**SF9 SEARCH**					
**Accession**	**Description**	**# PSMs**	**# AAs**	**MW [kDa]**	**calc. pI**
I7FV58	Ubiquitin OS=Spodoptera frugiperda PE=2 SV=1 - [I7FV58_SPOFR]	1	76	8.6	7.25
Q8WQI5	40S ribosomal protein S8 OS=Spodoptera frugiperda GN=RpS8 PE=2 SV=1 - [RS8_SPOFR]	1	208	23.8	10.64
C8CJY7	Triosephosphate isomerase (Fragment) OS=Spodoptera frugiperda GN=tpi PE=3 SV=1 - [C8CJY7_SPOFR]	1	105	11.4	5.22
G3CKA6	Actin OS=Spodoptera frugiperda PE=1 SV=1 - [G3CKA6_SPOFR]	4	376	41.7	5.48
Q95V36	Ribosomal protein S3 OS=Spodoptera frugiperda PE=2 SV=1 - [Q95V36_SPOFR]	1	243	26.8	9.69
G3CKA7	Alpha-tubulin OS=Spodoptera frugiperda PE=2 SV=1 - [G3CKA7_SPOFR]	1	450	49.9	5.19
Q8ISR0	Beta-1 tubulin (Fragment) OS=Spodoptera frugiperda PE=2 SV=1 - [Q8ISR0_SPOFR]	1	56	6.8	4.5
A0A0K2CTM7	Heat shock protein 70 A1 (Fragment) OS=Spodoptera frugiperda PE=2 SV=1 - [A0A0K2CTM7_SPOFR]	4	178	19.5	8.07
Q6B816	Histone H4 (Fragment) OS=Spodoptera frugiperda PE=4 SV=1 - [Q6B816_SPOFR]	1	43	4.9	11.31
Q8I866	Heat shock cognate 70 protein OS=Spodoptera frugiperda PE=2 SV=1 - [Q8I866_SPOFR]	6	659	73.1	5.34
Q9GQG6	90-kDa heat shock protein HSP83 OS=Spodoptera frugiperda GN=hsp83 PE=2 SV=1 - [Q9GQG6_SPOFR]	1	717	82.5	5.07
X4ZE67	Actin-related protein Arp2 (Fragment) OS=Spodoptera frugiperda GN=ARP2 PE=2 SV=1 - [X4ZE67_SPOFR]	1	394	44.9	6.16
**BACULOVIRUS SEARCH**					
**Accession**	**Description**	**# PSMs**	**# AAs**	**MW [kDa]**	**calc. pI**
A0A097PV47	AcOrf-81 OS=Autographa californica nuclear polyhedrosis virus GN=AcOrf-81 PE=4 SV=1 - [A0A097PV47_NPVAC]	1	220	25.5	9.03

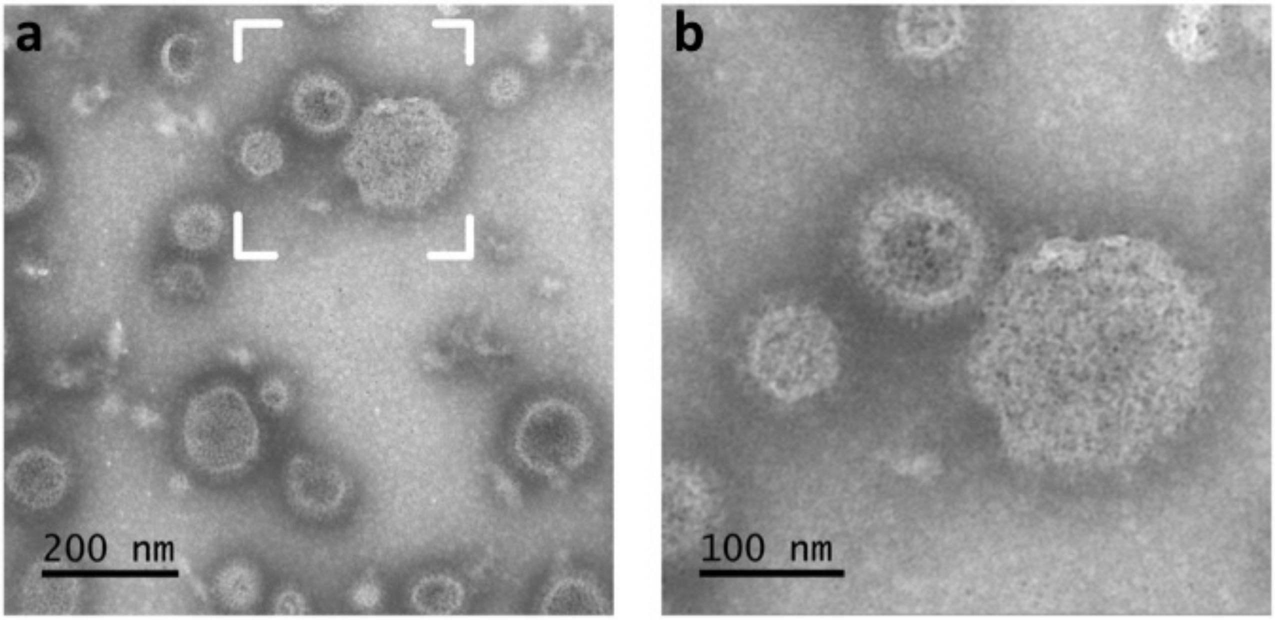

Figure S3

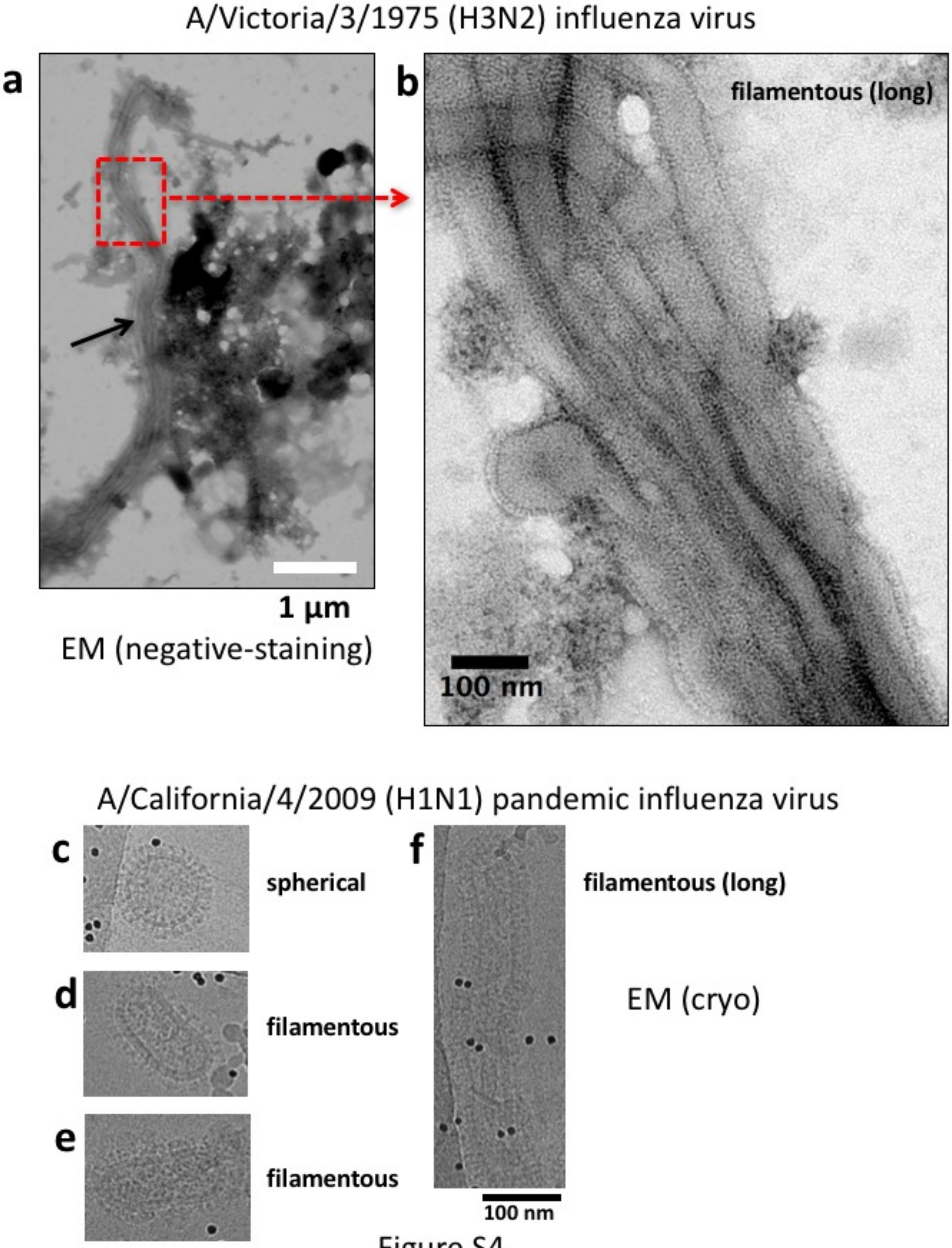
A/Victoria/3/1975 (H3N2) influenza virus
a
b
filamentous (long)
1 µm
EM (negative-staining)
100 nm
A/California/4/2009 (H1N1) pandemic influenza virus
c
spherical
d
filamentous
e
filamentous
f
filamentous (long)
EM (cryo)
100 nm

Figure S4

31

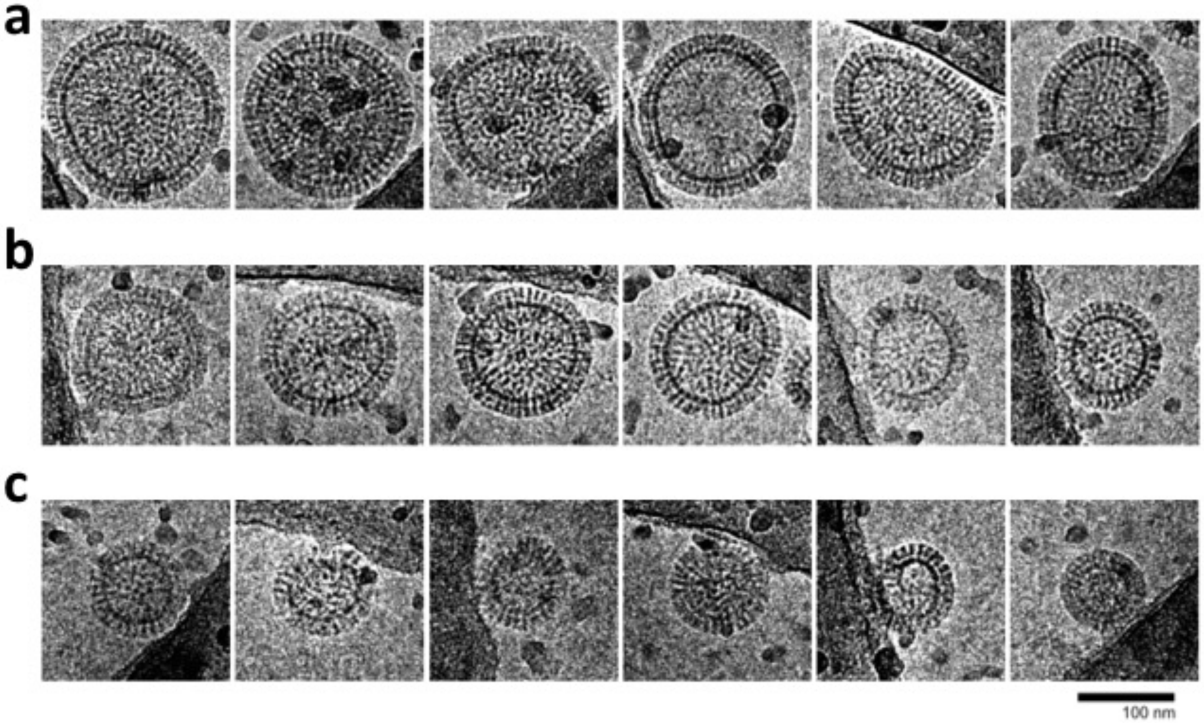

Figure S5

32

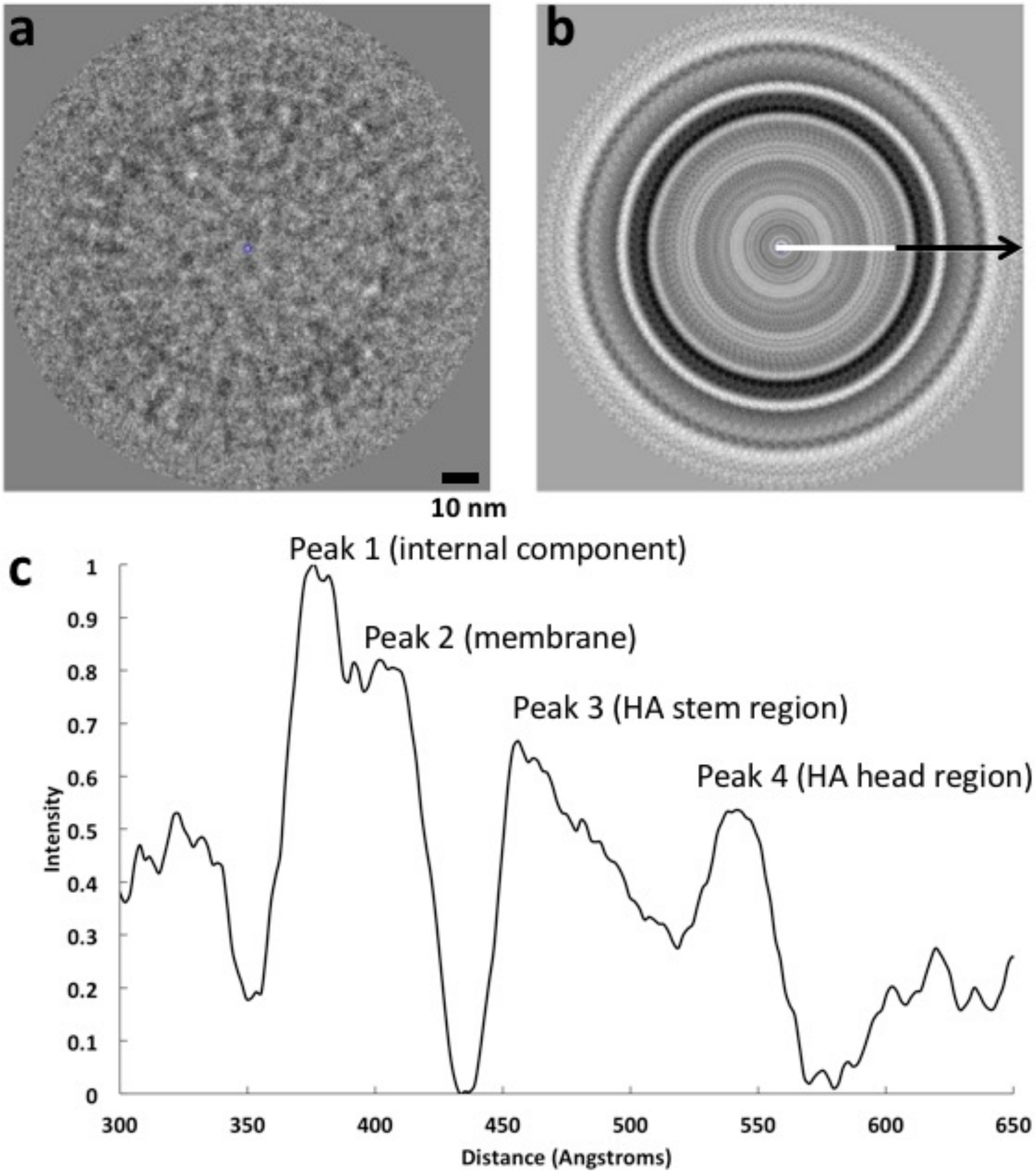
a
b
10 nm
c
Peak 1 (internal component)
Peak 2 (membrane)
Peak 3 (HA stem region)
Peak 4 (HA head region)
Intensity
Distance (Angstroms)
0
0.1
0.2
0.3
0.4
0.5
0.6
0.7
0.8
0.9
1
300
350
400
450
500
550
600
650

Figure S6

33

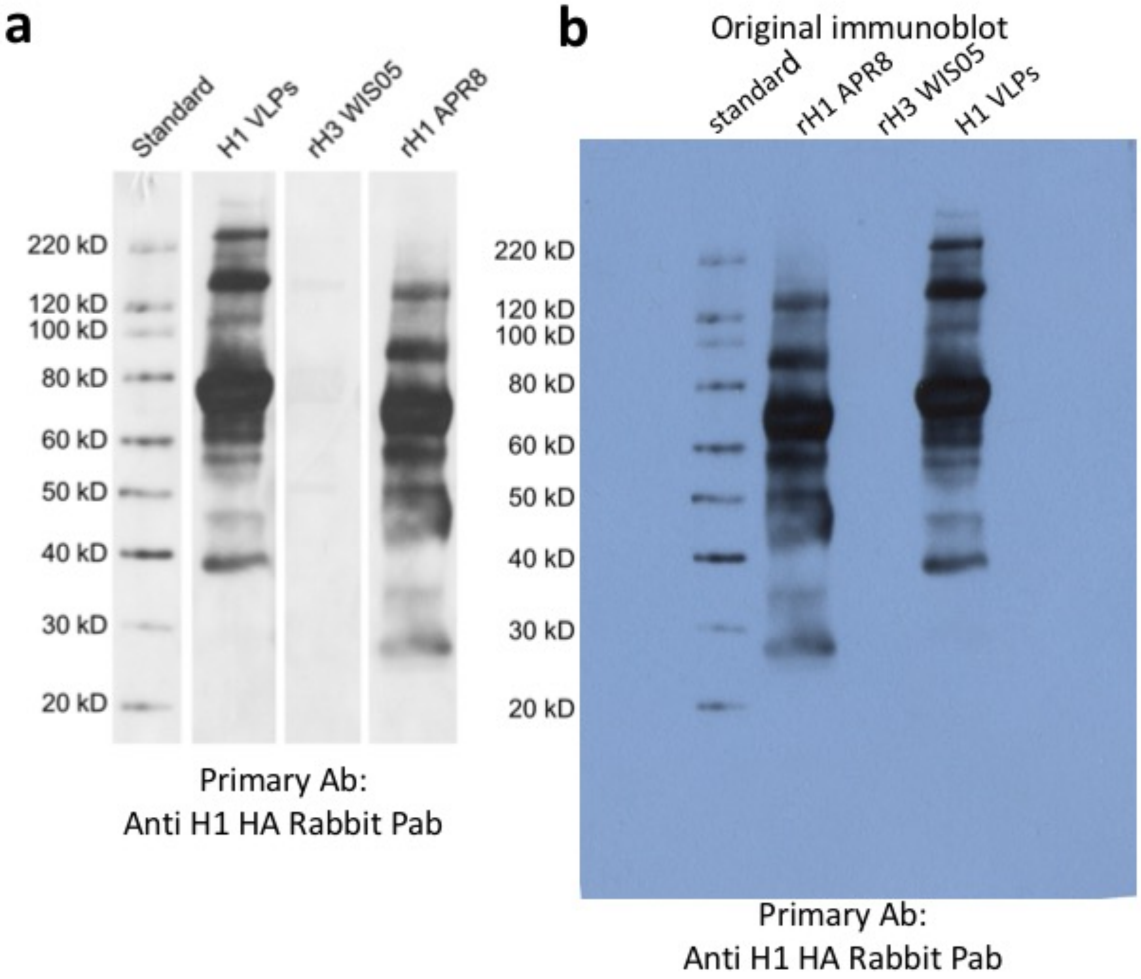
a
Standard
H1 VLPs
rH3 WIS05
rH1 APR8
220 kD
120 kD
100 kD
80 kD
60 kD
50 kD
40 kD
30 kD
20 kD
Primary Ab:
Anti H1 HA Rabbit Pab
b
Original immunoblot
standard
rH1 APR8
rH3 WIS05
H1 VLPs
220 kD
120 kD
100 kD
80 kD
60 kD
50 kD
40 kD
30 kD
20 kD
Primary Ab:
Anti H1 HA Rabbit Pab

Figure S7

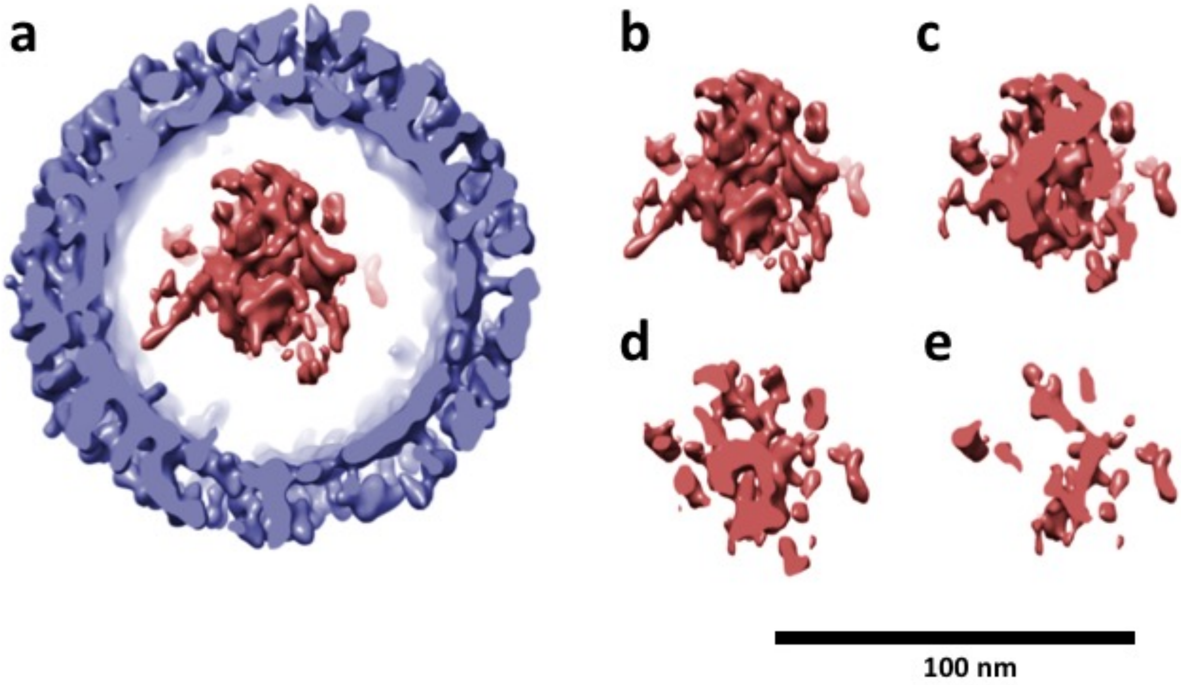

Figure S8

35

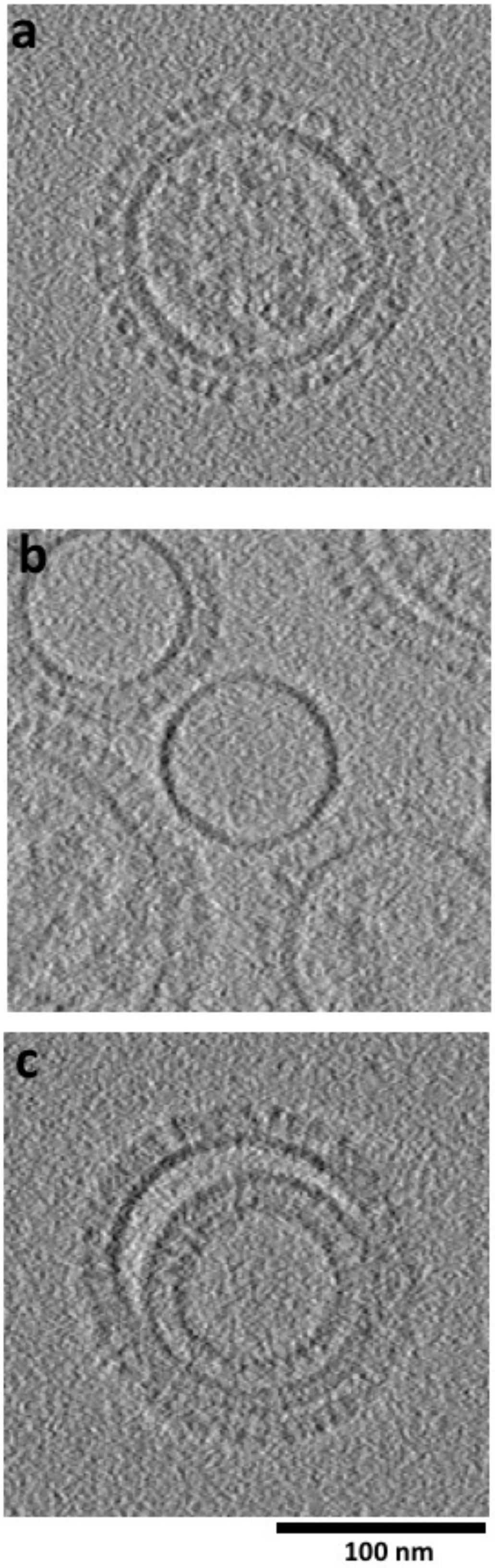

Virus-like particle (VLP)

Vesicle (surrounded by VLPs)

VLP (with internal vesicle with smaller VLP inside)

Figure S9

a

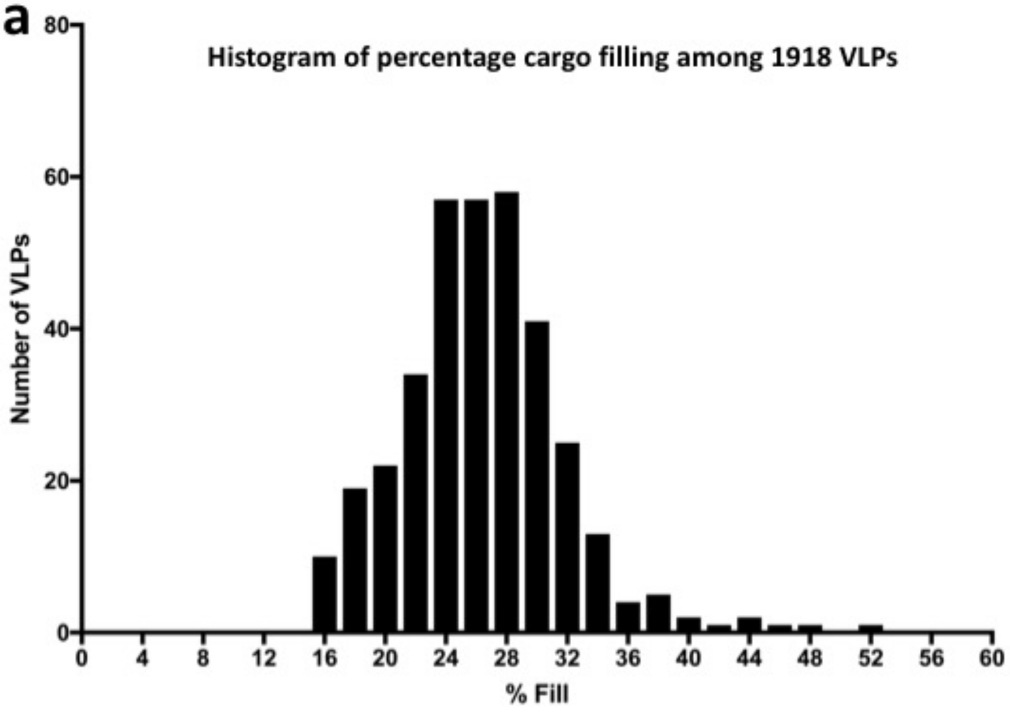

b

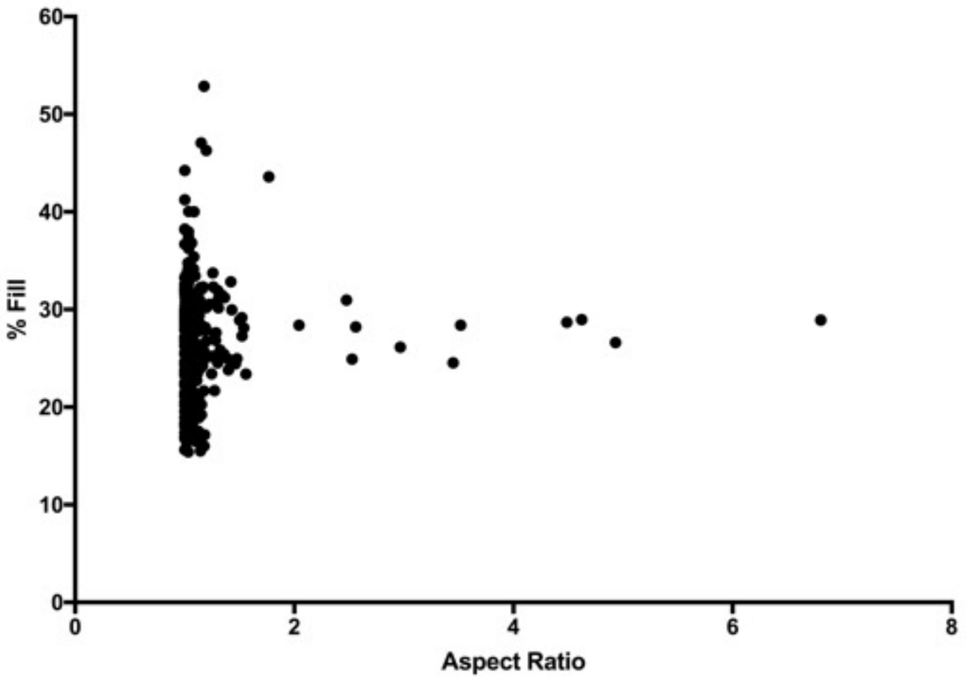

Figure S10

37

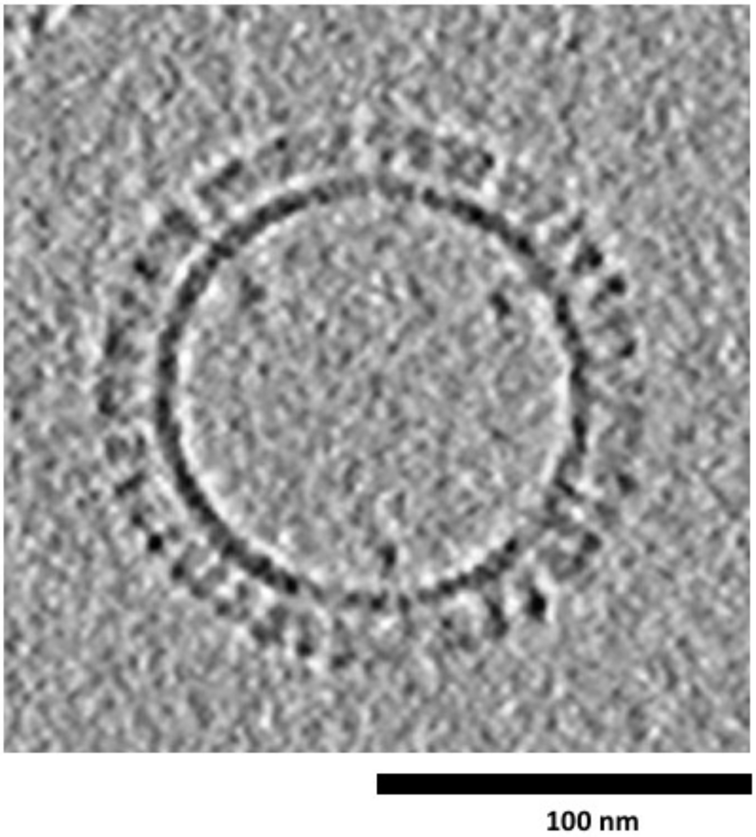

Movie S1

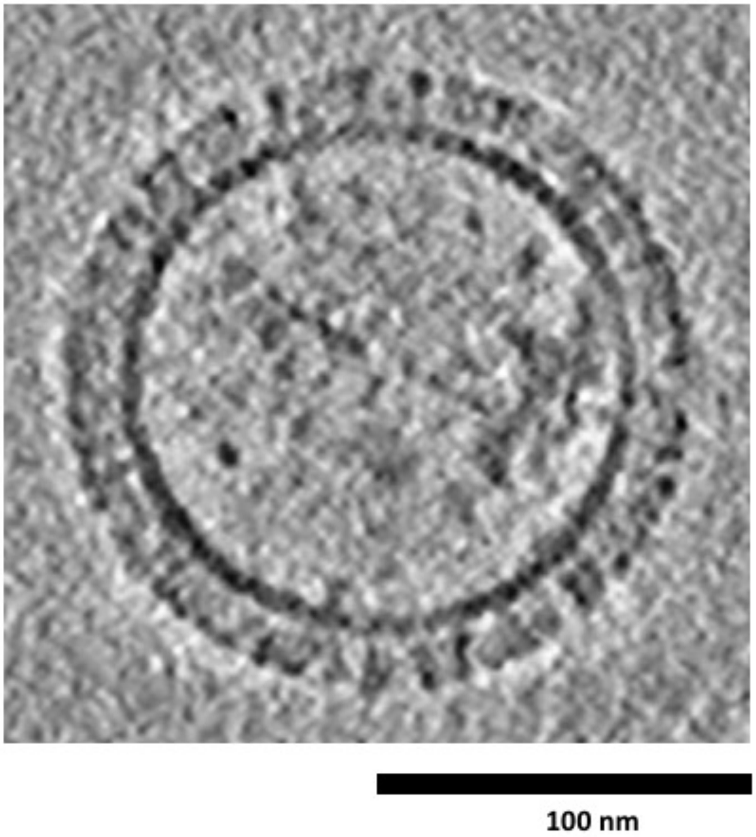

Movie S2

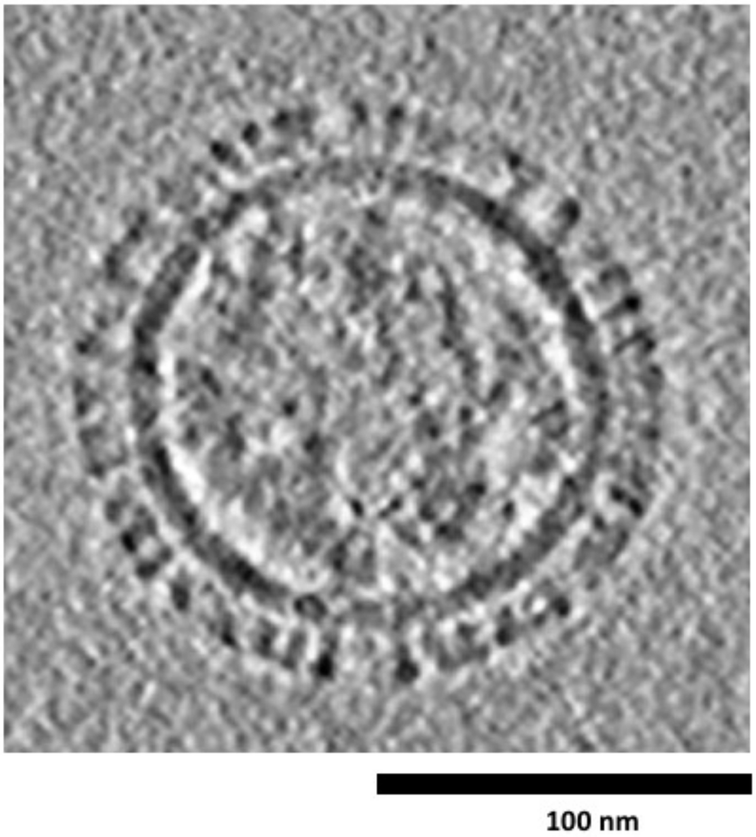

Movie S3

SUPPLEMENTAL FIGURE CAPTIONS

Figure S1. Analysis of composition and purity of hemagglutinin in 1918 influenza virus-like particles (VLPs). (a) SDS-PAGE analysis of VLPs under reducing conditions. Samples are molecular weight standards (std) and VLPs. A major band at an apparent molecular weight of about 75 kilodaltons is in the VLP sample. The major bind is indicated by an arrow. (b) 1-D density profile of protein standards with molecular weights as labeled. (c) 1-D density profile of 1918 influenza VLP sample from SDS-PAGE in panel a. Areas under peaks are in red. Relative abundance was calculated as the area under the peak above the baseline as compared to other background areas. The relative abundance for each peak is as labeled. The hemagglutinin is the dominant component of VLPs (greater than 91%).

Figure S2. Analysis of structure and composition of H1N1 influenza virus (A/Puerto Rico/8/1934 (H1N1)). (a) Image by cryo-electron microscopy of a field of virus particles with spherical and filamentous morphologies. Some spherical (black arrows) and filamentous virions (white arrows) are indicated. (b) SDS-PAGE analysis of virus with molecular weight standards (std) and PBS. Major bands of structural proteins are at apparent molecular weights of about 75 kDa (hemagglutinin, HA), 56 kDa (nucleoprotein, NP) and 27kDa (matrix, M1) in the virus sample. (c) (Top panel) 1-D profile of protein standard with molecular weights as labeled and (bottom panel) 1-D profile of influenza virus bands from SDS-PAGE. (d, e, f). Near central slices through 3D tomographic volumes of influenza virions with spherical morphologies. (g, h, i) Near central slices through 3D tomographic volumes of influenza virions with filamentous morphologies. Panels d to i are on the same scale. Scale bars, 100 nm.

Figure S3. Analysis of virus-like particles by negative-staining electron microscopy. (a) Electron microscopy images of 1918 VLPs negatively stained with 1.5% phosphotungstic acid at 52,000x magnification. (b) Higher magnification (110,000x) of the bracketed region in panel a. Spikes are observed on the surface. Image contrast is with protein as white. Scale bars, 200, 100 nm.

Figure S4. Filamentous morphologies of influenza viruses as observed by electron microscopy. (a) Negative-staining electron microcopy of a field of long filamentous influenza viruses (A/Victoria/3/1975 (H3N2) grown in MDCK cells. Scale bar, one micrometer. (b) Higher magnification of the region boxed in panel a. Several filamentous particles are close together and are hundreds of nanometers in length. Scale bar, 100 nm. (c, d, e, f) Cryo-electron microscopy of 2009 pandemic influenza viruses (A/California/4/09 (H1N1)) grown in embryonated chicken eggs. (c) Virus with spherical morphology. (d, e) Viruses with filamentous morphologies. (f) Virus with long filamentous morphology. Panels c to f are on the same scale. Scale bar for panels c-f, 100 nm.

Figure S5. Montage of examples of individual virus-like particles (VLPs) as observed by cryo-electron microscopy. VLPs are arranged in order of largest to smallest. (a) Larger-, (b) medium-, (c) smaller-sized particles. Most VLPs possessed a spherical or near-spherical morphology, but the overall size of VLPs is variable. Image contrast is with protein as black. Panels are on the same scale. Scale bar, 100 nm.

Figure S6. Analysis of density distribution of a virus-like particle (VLP). (a) Image of a spherical VLP by cryo-electron microscopy. Scale bar, 10 nm. (b) 1D circular average of the VLP. An arrow denotes the direction of the profile trace with the arrow as white for the inside and with the arrow as black close to the membrane and glycoprotein region used in subsequent 1D profile analysis. Image contrast is black. (c) 1D density profile of the circular average from the black arrow region in panel b. The major peak regions are labeled with corresponding interpreted assignments to internal, membrane and hemagglutinin components.

Figure S7. Reactivity of 1918 H1 hemagglutinin (HA) virus-like particles with antibodies by immunoblot. Samples were probed for reactivity with an anti-H1 rabbit polyclonal as the primary antibody. (a) Immunoblot with lanes from original immunoblot (panel b) separated into separate panels for clarity. Samples were 1918 H1 HA VLPs, recombinant H3 HA (A/Wisconsin/67/05 (H3N2) and recombinant H1 HA (A/PR/8 (H1N1). Major bands at ~75kDa were detected for H1

VLPs and recombinant H1 HA protein (positive control) while recombinant H3 HA (negative control) was not reactive. The anti-H1 rabbit polyclonal antibody bound strongly to H1N1 subtypes, but not to the H3N2 subtype. (b) Original immunoblot without lanes separated into individual panels.

Figure S8. Analysis of molecular regions of a virus-like particle (VLP) by segmentation of the tomographic volume. (a) Segmented model of a VLP. The membrane and hemagglutinin layers are colored in light blue and the internal component is colored in brick red. (b-e) The internal component is shown alone with slices through the volume by increasing z-clippings. Panels are on the same scale. Scale bar, 100 nm.

Figure S9. Comparison of virus-like particle (VLP) versus membrane vesicle by cryo-tomography. (a) Slice through a tomographic volume of a VLP. (b) Vesicle surrounded by neighboring VLPs. (c) A VLP with an apparent vesicle inside. The internal vesicle appears to have a smaller VLP on the inside. VLPs have spikes on the surfaces while vesicles appear smooth and spike-less. Vesicles were less than 1% of the particles observed. Panels are on the same scale. Scale bar, 100 nm.

Figure S10. Semi-automated analysis of influenza virus-like particles (VLP) tomograms in terms of relative amounts of internal density (% fill) and relationship to VLP aspect ratio. (a) Histogram of the relative amount of internal density filling each VLP. The distribution of the cargo has a peak at 28%, with the average molecular cargo fill being 26.4 $\pm$ 5.4%. (b) Plot of the aspect ratio of the VLPs against the % fill. VLPs were modeled as ellipses, and the aspect ratio was the length (nm) of major axis of the ellipse divided by length of minor axis. Automated analysis of VLP subtomograms computed the % fill of the molecular cargo for each of the 353 VLPs using a fixed density threshold. All VLPs had internal density (i.e. filled to some extent). While the morphology of the VLP was uncorrelated with the % fill, the amount of cargo was consistent with a random distribution centered about 26%.

SUPPLEMENTAL MOVIE CAPTIONS

Movie S1. Movie that slices through a cryo-electron microscopic 3D volume (tomogram) of a 1918 H1 HA virus-like particle that is categorized with relative low amounts of internal components (i.e. molecular cargo). Scale bar, 100 nm.

Movie S2. Movie that slices through a cryo-electron microscopic 3D volume (tomogram) of a 1918 H1 HA virus-like particle that is categorized with relative medium amounts of internal components (i.e. molecular cargo). Scale bar, 100 nm.

Movie S3. Movie that slices through a cryo-electron microscopic 3D volume (tomogram) of a 1918 H1 HA virus-like particle that is categorized with relative high amounts of internal components (i.e. molecular cargo). Scale bar, 100 nm.

SUPPLEMENTAL METHODS

Semi-automation of VLP tomogram analysis for internal density

VLP tomograms were cropped to display a 10 nm central slab for each VLP using the Chimera package. Images were produced for each slab and the size and morphology of the VLPs was calculated as described for the size and morphology measurements. Ellipses containing the interior of the VLPs were analyzed using the Measure tool in Fiji. The % fill of molecular cargo for each slab was measured by thresholding the density at a fixed value, and then calculating the area containing cargo divided by the total area of the ellipse. In this case, internal VLP density computational classified as 'low' was less than 23% filled, medium between 23-29%, and high greater than 30%. Computational classification methods yielded 24% of VLPs classified as low, 42% as medium, and 34% as high, which is similar to visual classification methods and with the majority of VLPs having internal densities like visual classification. Both analyses of VLPs indicated that the majority of the VLPs contained internal densities.
